# Local, Sustained, and Targeted Co-Delivery of MEK Inhibitor and Doxorubicin Inhibits Tumor Progression in E-Cadherin-Positive Breast Cancer

**DOI:** 10.3390/pharmaceutics16080981

**Published:** 2024-07-25

**Authors:** Paul M. Kuhn, Gabriella C. Russo, Ashleigh J. Crawford, Aditya Venkatraman, Nanlan Yang, Bartholomew A. Starich, Zachary Schneiderman, Pei-Hsun Wu, Thi Vo, Denis Wirtz, Efrosini Kokkoli

**Affiliations:** 1 Department of Chemical and Biomolecular Engineering, Johns Hopkins University, Baltimore, MD 21218, USA; 2 Johns Hopkins Institute for NanoBioTechnology, Johns Hopkins University, Baltimore, MD 21218, USA; 3 Johns Hopkins Physical Sciences—Oncology Center, Johns Hopkins University, Baltimore, MD 21218, USA; 4 Department of Oncology, Johns Hopkins University School of Medicine, Baltimore, MD 21287, USA; 5 Department of Pathology, Johns Hopkins University School of Medicine, Baltimore, MD 21287, USA

**Keywords:** MEK inhibitor, doxorubicin, E-cadherin positive breast cancer, hydrogel, PR_b-targeted liposomes

## Abstract

Effectively utilizing MEK inhibitors in the clinic remains challenging due to off-target toxicity and lack of predictive biomarkers. Recent findings propose E-cadherin, a breast cancer diagnostic indicator, as a predictor of MEK inhibitor success. To address MEK inhibitor toxicity, traditional methodologies have systemically delivered nanoparticles, which require frequent, high-dose injections. Here, we present a different approach, employing a thermosensitive, biodegradable hydrogel with functionalized liposomes for local, sustained release of MEK inhibitor PD0325901 and doxorubicin. The poly(δ-valerolactone-*co*-lactide)-*b*-poly(ethylene-glycol)-*b*-poly(δ-valerolactone-*co*-lactide) triblock co-polymer gels at physiological temperature and has an optimal degradation time in vivo. Liposomes were functionalized with PR_b, a biomimetic peptide targeting the α_5_β_1_ integrin receptor, which is overexpressed in E-cadherin-positive triple negative breast cancer (TNBC). In various TNBC models, the hydrogel-liposome system delivered via local injection reduced tumor progression and improved animal survival without toxic side effects. Our work presents the first demonstration of local, sustained delivery of MEK inhibitors to E-cadherin-positive tumors alongside traditional chemotherapeutics, offering a safe and promising therapeutic strategy.

## 1. Introduction

Around 30% of human solid tumors are characterized by a mutation in the RAS gene, leading to aberrant activation of the RAS-RAF-MEK-ERK pathway [1]. MEK1/2 plays a crucial role in tumorigenesis, cell proliferation, and inhibition of apoptosis; thus, MEK inhibition is an attractive therapeutic strategy in numerous cancers. In the past decade, several potent MEK1/2 inhibitors with high affinity for desired phosphosites have been developed as oral agents. However, because MAPK is a critical pathway in all cell types, systemic delivery is associated with off-target effects. Hence, MEK inhibitors have historically failed in the clinical setting due to prohibitive toxicity (i.e., narrow therapeutic index), demonstrating a need for predictive biomarkers [2]. Clinical trial results validate the idea that biomarkers can aid in improving MEK inhibitor efficacy. There are only three FDA-approved MEK inhibitors, all for BRAF-mutated melanoma, as this mutation prevents adverse effects from off-target effects [2]. The high success observed in this subset of melanoma patients demonstrates the need for cancer-specific biomarkers to improve MEK inhibitor efficacy.

E-cadherin, a cell–cell adhesion molecule, has emerged as a potential new biomarker for indicating if a subtype of breast cancer will respond to MEK inhibition. Approximately half of all major worldwide institutions routinely assess E-cadherin (E-cad) expression in breast biopsies to classify invasive ductal and lobular carcinoma [3,4]. Invasive ductal carcinoma (IDC) accounts for 80% of all breast cancers, 90% of which are E-cad positive [5]. Significant evidence for the role of E-cad in tumor progression and metastatic potential of tumor cells justifies a reclassification of E-cad as an oncogene, making it an excellent biomarker candidate for specific cancer therapies. In breast cancer specifically, recent studies have demonstrated that E-cad is surprisingly required for effective metastasis and is responsible for rapid growth of primary tumors and metastatic outgrowth [6,7,8].

E-cad expression correlates with worse overall survival in breast cancer patients, further confirming that its classification as a tumor suppressor gene is no longer accurate (Figure S1a). Russo et al. recently demonstrated that E-cad interacts with EGFR, which results in increased activation of the MEK/ERK cascade in the MAPK signaling pathway, causing a highly proliferative and invasive phenotype in several models of IDC [7]. This aggressive tumor cell phenotype can be combatted with small-molecule inhibitors that prevent MEK/ERK activation and the consequential downstream effects of ERK that result in hyper-proliferation. One such MEK inhibitor, PD0325901 (mirdametinib), can revert the hyper-proliferative phenotype induced by E-cad expression, both in vitro and in vivo, in various subtypes of IDC, including triple negative breast cancer (TNBC) and hormone receptor-positive (HR+) breast cancer [7]. However, clinically translating this finding remains a challenge. Although there is ongoing work to improve the efficacy of MEK inhibitors by combining them with chemotherapeutics, the results have been inconclusive, and these trials ultimately fail from lack of benefit and/or adverse effects [2]. Currently, the inclusion of MEK inhibitors in a chemotherapeutic regime for breast cancer patients is not feasible with systemic delivery.

Recent work utilizing nanoparticles to deliver MEK inhibitors and cytotoxic agents with nanoparticles have shown promising results [9,10]. The co-delivery of MEK inhibitor AZD6244 and cisplatin in hyaluronic acid-coated oleic acid nanoparticles to target CD44 was able to achieve significant toxicity in colon cancer when tested in vitro [10]. The delivery of another MEK inhibitor, PD98059, in polylactic acid glycolic acid (PLGA) nanoparticles could improve the efficacy of cisplatin delivered via intraperitoneal (i.p.) injection in murine melanoma tumors [11]. The combination of an MEK inhibitor delivered via nanoparticles with i.p. cisplatin ablated 50% of tumors completely compared to none when free-form MEK inhibitor was given via i.p. injection. Other strategies to improve MEK inhibitor efficacy include combinations with various other classes of inhibitors to create blockades [12,13,14,15]. Layer-by-layer liposomes were used to deliver both MEK and PI3K inhibitors in an attempt to generate a horizontal blockade and prevent crosstalk between pathways, which had been hypothesized to reduce MEK inhibitor efficacy [11,16]. Taken together, these results suggest that employing targeted nanoparticles is a promising approach to improving MEK inhibitor efficacy. However, systemic delivery of nanoparticles has limitations, as most of the dosage is cleared. Recent efforts have focused on adding targeting mechanisms to increase nanoparticle uptake at the tumor site with promising results, yet frequent and high dosage is still required [11,12,13,14,15,16].

To overcome the current limitations of MEK inhibitors, we first employed targeted nanoparticles to reduce off-target effects and improve therapeutic efficacy. We generated targeted nanoparticles by decorating the surface of liposomes with PR_b, a fibronectin mimetic peptide that binds specifically to α_5_β_1_ integrin with high affinity [17,18]. Binding and internalization of PR_b functionalized liposomes depend on the surface expression of α_5_β_1_ integrin [17,19]. Thus, PR_b functionalized liposomes have been previously utilized to deliver therapeutics in several cancer types in which integrin is overexpressed in vitro and in vivo [17,20,21,22]. Although targeted nanoparticles are an attractive delivery system due their ability to enhance uptake at the tumor site, less than 10% of the injected dose makes it to the solid tumor after systemic injection [23].

To overcome the limitation of systemic delivery, we designed a novel platform designed to achieve localized, sustained delivery of targeted nanoparticles via encapsulation of liposomes in a thermosensitive and biodegradable hydrogel. Thermosensitive hydrogels are an appealing drug delivery system (DDS), as most are liquid at room temperature, allowing for nanoparticles to be directly mixed with polymeric nanoparticles and injected, eliminating the need for surgical implantation [24,25]. Thus, the hydrogel forms a drug depot system, providing sustained release of drugs directly to the tumor site [26]. Here, we utilize a poly(δ-valerolactone-*co*-lactide)-*b*-poly(ethylene-glycol)-*b*-poly(δ-valerolactone-*co*-lactide) (PVLA-PEG-PVLA) triblock copolymer, which undergoes a spherical-to-wormlike micelle transition at physiological temperature (37 °C) that has been shown to cause gelation [27]. Thus, once injected into the body, the PVLA-PEG-PVLA hydrogel forms a semi-solid structure that entraps the nanoparticles in the local tumor environment. The hydrogel achieves an optimal balance between degradation time and pH stability, while other thermosensitive hydrogels require high temperatures (≥60 °C) or high salt concentrations to achieve the unique phase transition to wormlike micelles [28,29]. Our DDS is an ideal platform for the delivery of chemotherapeutics directly to solid tumors, avoiding systemic administration and frequent injections [21].

In this work, we employ both targeted nanoparticles and a thermosensitive, biodegradable hydrogel to improve the efficacy of MEK inhibitors when given in combination with chemotherapeutics. The proposed DDS was studied extensively in vitro utilizing a multi-compartment tumor organoid model [30], which confirmed increased nanoparticle uptake with PR_b peptide and sustained release with hydrogel encapsulation. In pre-clinical models of E-cad-positive IDC, we demonstrated the potential clinical translatability of co-delivering iMEK and DOX in our DDS and determined if the hydrogel itself elicits an immune response. This approach aims to provide a solution to both the prohibitive toxicity of MEK inhibitors and the need for a predictive biomarker.

## 2. Materials and Methods

### 2.1. Materials

D,L-lactide was purchased from Acros Organics (Geel, Belgium); δ-valerolactone was purchased from Alfa Aesar (Haverhill, MA, USA); and stannous octoate, Sephadex G-50, and calcein were purchased from Sigma-Aldrich (Milwaukee, WI, USA). Dipalmitoylphosphatidylcholine (DPPC), cholesterol, and 1,2-dipalmitoyl-sn-glycero-3-phosphoethanolamine-*N*-[methoxy(polyethylene glycol)-750] (DPPE-PEG750) (ammonium salt) were purchased from Avanti Polar Lipids (Birmingham, AL, USA). Polyethylene glycol 1500 (PEG1500) was purchased from Millipore Sigma (St. Louis, MO, USA), and PrestoBlue assay was purchased from Thermo Fisher Scientific (Waltham, MA, USA). All organic solvents (HPLC grade) were purchased from Sigma-Aldrich (Milwaukee, WI, USA) and Fisher Scientific (Waltham, MA, USA). Items used for culturing cells were purchased from Corning (Corning, NY, USA) and Sarstedt (Newton, NC, USA). All other chemicals and materials were purchased from Sigma-Aldrich.

### 2.2. Synthesis of PVLA-PEG-PVLA Triblock Copolymer

PVLA-PEG-PVLA triblock copolymer synthesis utilizing bulk ring-opening polymerization of δ-valerolactone and D,L-lactide was previously published, with full material characterization [27]. Briefly, δ-valerolactone was vacuum-distilled to remove the polymerized monomer, and D,L-lactide was recrystallized in ethyl acetate three times overnight. Simultaneously, PEG1500 was purified by vacuum-drying at 50 °C overnight. For polymer synthesis, 10.94 g of PEG1500 was first transferred to a Schlenk flask, followed by 5.10 g of δ-valerolactone and 20.41 g of D,L-lactide. Stannous octoate (0.02 g/mL) was then added to the flask as the catalyst. After reacting the components for 14 h at 130 °C under an argon atmosphere, the reaction mixture was cooled to room temperature. It was then dissolved in dichloromethane and precipitated in ice-cold ethyl ether. The precipitate was dried in a vacuum oven at room temperature for 3 days and stored at −20 °C until further use.

### 2.3. Cryo-TEM Studies

The morphologies of self-assembled polymer nanoparticles were observed with cryo-TEM at various temperatures. To prepare the samples, polymer solution in water was prepared at 20 *w*/*v*% and then further diluted to 0.25 *w*/*v*%, followed by heating to 25 °C and 37 °C for 1 h using a dry bath. Cryo-TEM grids (Ted Pella, Redding, CA, USA) were glow-discharged for 60 s at 1 mA setting, followed by sample deposition and vitrification in liquid ethane by Vitrobot using the following parameters: 3 s blot time, 0 offset, 3 s wait time, 0 s drain time, 0 blot force, and 100% relative humidity. The prepared sample grids were stored under liquid nitrogen until they were transferred to a F200C Talos TEM operated with an acceleration voltage of 200 kV (Integrated Imaging Center, Johns Hopkins Institute for NanoBioTechnology), and images were acquired using a Ceta camera. 

iMEK+DOX-loaded liposomes were also examined with cryo-TEM. Approximately 5 μL of aqueous suspension of liposomes at 9 mM lipids was deposited onto carbon copper grids and vitrified in liquid ethane by Vitrobot using the following parameters: 2 s blot time, 0 offset, 0 s wait time, 0 s drain time, 0 blot force, and 100% relative humidity. The prepared samples were then stored and imaged as mentioned above.

### 2.4. Coarse Grain Molecular Simulation

PVLA particles were assigned an attractive Lennard–Jones (LJ) interaction with each other [31]. This captured the hydrophobic interactions that drive PVLA segregation from the surrounding water. PEG–PEG and PVLA–PEG interactions were both repulsive, modeled using the Weeks–Chandler–Andersen (WCA) potential [31]. Specifically, PEG–PEG repulsions were defined to decrease with increasing temperature to mimic the effect of PEG hydration from the surrounding water. PEG–PEG repulsion captured the formation of a water solvation shell around PEG monomers, which drove swelling of the PEG block. Increasing temperature produced PEG dehydration, causing shrinkage of the PEG block, and was captured in our model via a weakening of PEG–PEG repulsion. PEG dehydration also decreased solvent screening between PVLA and PEG, which would increase the segregating force between the two polymeric blocks. Analogous to our approach for modulating PEG–PEG interactions, PVLA–PEG interactions were defined to increase with increasing temperature to capture the stronger segregation. Parameters of interest that were varied in our MD simulations were the system temperature and ratio of PVLA-to-PEG chain lengths. For details, we refer the reader to the Supplementary Materials.

### 2.5. Scaling Theory for Spherical-to-Wormlike Micelle Transition

To generate the phase diagram, we derived a scaling theory that aimed to capture the spherical-to-wormlike micelle transition for the BAB triblock copolymer system. Our theory operated under the following two assumptions: (1) the B block (PVLA) forms the interior of the micelle and (2) the A block (PEG) forms the exterior of the micelles and behaves analogously to a “grafted” layer to the B block core. In other words, the physical picture is that of a spherical core of size $R_{B}$ formed by the aggregated B block, covered by a “grafted” layer consisting of A block chains.

The size of the A block layer, $R_{A}$, could be approximated using our previously derived scaling theory for grafted chains in a good solvent [32,33,34]:

(1) $$R_{A}\;\sim \;f^{1/5}v_{A}^{1/5}b_{A}N_{A}^{3/5}$$

where $N_{A}$ is the degree of polymerization of the A block, f is the number of chains anchored to the core, and $v_{A}$ and $b_{A}$ are the excluded volume and Kuhn length of the A-block monomer, respectively. Here, we note that f is equal to the aggregation number of BAB chains that form each micelle since the number of A blocks making up the corona must be equal to the number of chains within the entire structure. 

The size of the B block core depends on the local concentration of B chains present within the core. The transition from spherical to wormlike micelles is reminiscent of a concentration dependent theta to poor solvent transition, which can be captured via the following relationship:

(2) $$R_{B}\;\sim \;N_{B}^{1/3}b_{B}{\left[\frac{v_{B}\phi_{B}^{5/2}}{b_{B}^{3}}\right]}^{-1/3}$$

where $\phi_{B}$ is the concentration of the B block within the core region and all other terms are have analogous definitions to their A block counterparts.

Prediction of the spherical-to-wormlike micelle transition requires a balance between the free energy of the A block versus B block using the classical Flory-type theory [35]: F ~R2Nb2+vfN2R3, which requires knowledge of the aggregation number, f, of the number of chains making up each micelle. To determine f, we note that $R_{B}\;\sim \;fR_{A}$: that is, the aggregation number, f, is defined by the number of A blocks that can occupy the surface of the B block core. Plugging in $R_{A}$ and $R_{B}$, followed by solving for f, gives:

(3) $$f\;\sim \;N_{B}^{5/3}N_{A}^{-3}{\left(\frac{b_{B}}{b_{A}}\right)}^{5}{\left(\frac{v_{B}\phi_{B}^{5/2}}{b_{A}^{3}}\right)}^{-5/3}v_{A}$$

Combining the derived relationship for f with the Flory energy, F, we can then write the free energy of both the A block and B block to be the following:

(4) $$F_{B}\;\sim \;N_{B}^{-1/3}{\left(\frac{v_{B}\phi_{B}^{5/2}}{b_{B}^{3}}\right)}^{-2/3}\left[1+{\left(\frac{N_{B}}{N_{A}}\right)}^{3}{\left(\frac{b_{B}}{b_{A}}\right)}^{5}\left(\frac{v_{B}}{b_{B}^{3}}\right)v_{A}\right]$$

(5) $$F_{A}\;\sim \;N_{B}^{-1/3}{\left(\frac{v_{B}\phi_{B}^{5/2}}{b_{B}^{3}}\right)}^{-2/3}\left(\frac{N_{B}}{N_{A}}\right){\left(\frac{b_{B}}{b_{A}}\right)}^{2}v_{A}^{4/5}\left[1+b_{A}\right]$$

Defining the cross-over condition, $F_{A}\;\sim \;F_{B}$, provides the phase boundary between spherical and wormlike micelles:

(6) $$1+{\left(\frac{N_{B}}{N_{A}}\right)}^{3}{\left(\frac{b_{B}}{b_{A}}\right)}^{5}\left(\frac{v_{B}}{b_{B}^{3}}\right)v_{A}\;\sim \left(\frac{N_{B}}{N_{A}}\right){\left(\frac{b_{B}}{b_{A}}\right)}^{2}v_{A}^{4/5}\left[1+b_{A}\right]$$

Approximating $b_{A}\;\sim \;b_{B}$ simplifies Equation (6) to:

(7) $$\frac{v_{B}}{b_{B}^{3}}\;\sim \left[\left(\frac{N_{B}}{N_{A}}\right)v_{A}^{4/5}-1\right]{\left(\frac{N_{B}}{N_{A}}\right)}^{-3}v_{A}^{-1}$$

Note that vBbB3 is also the dimensionless excluded volume (${\tilde{v}}_{B}$) of each B monomer. This can be written as ${\tilde{v}}_{B}\;\sim \;{\tilde{v}}_{o}[1-2\chi ]$, where ${\tilde{v}}_{o}$ is a reference excluded volume and χ is the Flory–Huggins interaction parameter. Additionally, χ is empirically related to temperature via $\chi \;\sim \;G+HT^{-1}$, were G and H are constants. Combining these definitions with Equation (7) gives:

(8) $${\tilde{v}}_{o}\left[C-\frac{D}{T}\right]\;\sim \left[\left(\frac{N_{B}}{N_{A}}\right)v_{A}^{4/5}-\left(1+b_{A}\right)\right]{\left(\frac{N_{B}}{N_{A}}\right)}^{-3}v_{A}^{-1}$$

Equation (8) defines the phase boundary shown in the main text in Figure 2e.

### 2.6. Preparation and Characterization of Liposomes

The thin film hydration technique was used to prepare the liposomes. DPPC, cholesterol, and DPPE-PEG_750_ were dissolved in chloroform and mixed in the molar ratio of 64:35:1 [20,21,22]. The lipid mixture in a round bottom flask was fixed in a rotary evaporator at 50 °C to remove organic solvent. The film was vacuum-dried overnight at room temperature. The lipid film was rehydrated in 300 μM sulfo-Cy5 dye in PBS (pH 7.4) for 1 h at 65 °C for in vitro cell uptake studies. The vesicles were subjected to extrusion through 100 nm polycarbonate membranes for 21 cycles at 65 °C. The unencapsulated dye was removed by gel permeation chromatography using a Sephadex G-50 prepacked column. 

PD0325901 (iMEK) (Selleckchem (Houston, TX, USA), S1036)-loaded liposomes were prepared in a similar manner, where iMEK solution in chloroform was added to the lipid mixture at varying concentrations. The unencapsulated iMEK was removed by filtering the liposome suspension through a 0.45 μm polyvinylidene fluoride (PVDF) membrane filter. Doxorubicin HCl (DOX) (Selleckchem, S1208) was loaded in the liposomes at different concentrations by first rehydrating the lipid film with 1 mL of 250 mM ammonium sulfate solution in Milli-Q water for 1 h at 65 °C, followed by extrusion as described above and dialysis at 4 °C for 1.5 h to remove ammonium sulfate. The liposomal suspension was then mixed with a stock solution of DOX in HEPES (Quality Biological, Gaithersburg, MD, USA; 118-089-721) for 3 h at 65 °C. Unencapsulated DOX was removed through dialysis at 4 °C overnight. 

To assess the encapsulation efficiency of iMEK and DOX, the drug-loaded liposomes were ruptured with ethanol to make a 50:50 water/ethanol solution and centrifuged to pellet down the undissolved lipids and obtain a clear supernatant. The supernatant was analyzed using UV–Vis spectroscopy, carried out on a Spectramax M3 plate reader (Molecular Devices, San Jose, CA, USA). iMEK was detected at 275 nm and DOX at 485 nm, and their concentrations were determined using calibration curves.

PR_b-functionalized liposomes were prepared using a PR_b peptide-amphiphile synthesized as previously described [18] and added to the lipid mixture at 5 mol% initial concentration. The lipid concentration was determined using a Stewart assay, and the final concentration of the peptide on the liposome surface was determined using the BCA protein assay (Thermo Fisher Scientific, 23225), following the manufacturer’s protocol. The actual peptide concentration on the surface of the PR_b-functionalized liposomes was 3.4 ± 0.5 mol%. The particle size and zeta potential of the liposomes were determined using a Zetasizer (Malvern Panalytical, Malvern, UK). 

### 2.7. Preparation of Hydrogel Encapsulating Liposomes

A polymer solution of PVLA-PEG-PVLA in 1 mM HEPES was prepared at a concentration of 29 *w*/*v*% at 4 °C. HEPES buffer was added, and the pH of the solution was titrated to 7.4 with 1 M NaOH, resulting in a final gel concentration of 26.4 *w*/*v*%. Liposomes loaded with DOX/iMEK at a 2:1 molar ratio were mixed with the polymer solution at a 1:5 *v*/*v* ratio by stirring at 4 °C for 2 h. The resulting mixtures had a final polymer concentration of 22 *w*/*v*% and liposomes at 3 mM lipids for in vitro testing and 9 mM lipids for in vivo testing. The hydrogel nanoparticle mixtures were then placed in an incubator at 37 °C to complete the encapsulation. Gelation was observed in ~1 min, and gels were allowed to form for 1 h before use. 

### 2.8. Drug Release Studies

The release of drugs from hydrogels encapsulating iMEK/DOX-loaded liposomes was determined as previously described [21]. After preparing the hydrogel-nanoparticle system as discussed above, 2.5 mL of the mixture was placed in a dialysis tube (3500 Da MWCO) and allowed to gel at 37 °C for 1 h. The dialysis tubes were placed in 20 mL of release media (PBS, with 0.1% *v*/*v* Tween 80) and placed on an orbital shaker at 50 RPM. The release media was collected at different times and replaced with prewarmed fresh media. The collected samples were analyzed using UV–Vis spectroscopy. For the release of free drugs from the hydrogel, 22 *w*/*v*% polymer was combined with DOX dissolved in PBS and iMEK dissolved in PBS with 1% *v*/*v* Tween 80 for a final volume of 2.5 mL, and the experiment was performed as described above for the nanoparticle-hydrogel system. For the release of drugs from free liposomes in solution, the same protocol was followed, with 2 mL of liposome solution placed in the dialysis tube. 

### 2.9. Cell Culture

Human breast carcinoma cells MDA-MB-231 (ATCC) were cultured in Dulbecco’s Modified Eagle’s Medium (DMEM, Corning, Corning, NY, USA; 10-013-CV) supplemented with 10% (*v*/*v*) fetal bovine serum (FBS, Corning, Corning, NY, USA; 35-010-CV) and 1% penicillin-streptomycin (Gibco, Waltham, MA, USA; 15140-122). Healthy human breast epithelial cells 184B5 (ATCC) were cultured in Mammary Epithelial Cell Growth Medium (MEGM, Lonza, Walkersville, MD, USA; CC-3150). Mouse breast carcinoma cells 4T1-luc (ATCC) were cultured in RPMI-1640 (ATCC modification) medium (Thermo Fisher, Waltham, MA, USA; A1049101) supplemented with 10% (*v*/*v*) FBS and 1% penicillin-streptomycin. Cells were maintained at 37 °C and 5% CO_2_ in a humidified incubator during cell culture. To generate MDA-MB-231 E-cad knock-in cells, briefly, the lentiviral vector of E-cadherin-EGFP was generated from EGFP;pCS-CG (Addgene, Watertown, MA, USA; 12154) via cloning full-length E-cadherin upstream of EGFP between the Nhe 1 and Age 1 sites of pCS-CG to generate an EGFP-fused protein [36]. A scramble sequence was also inserted (Addgene, Watertown, MA, USA; 162011) and the scramble control line was used in all experiments for the E-cad cell line. Genetically modified cells were kept in puromycin selection during maintenance only.

### 2.10. Multi-Compartment Tumor Organoids

Organoid cores were generated with high-concentration Matrigel (Corning, Corning, NY, USA; 354248) at a density of 10,000 cells/μL. As previously described, 1 μL droplets of Matrigel/cell solution were made utilizing oil-in-water droplet technology [30,37,38,39]. Cores were then wrapped in 2 mg/mL collagen I gel, prepared as previously described [40]. Briefly, collagen I (Corning, Corning, NY, USA; 354249) was mixed with reconstitution buffer and titrated to pH 7.4. Then, 10 μL of collagen I matrix and a single Matrigel core were pipetted to generate the multi-compartment organoid. The PrestoBlue assay was used at different time points to determine the fold change in cell number over time [7,38].

### 2.11. Expression of α_5_β_1_ Integrin 

Expression of the α_5_β_1_ integrin receptor on MDA-MD-231 organoids was investigated using flow cytometry. Organoids were allowed to grow for 4 days before cells were harvested and analyzed. Briefly, the organoids were dissociated by first incubating in 20 μg/mL collagenase (Gibco, 17018029) for 30 min at 37 °C. The organoids were then washed twice with PBS and incubated in Cell Recovery Solution (Corning, Corning, NY, USA; 354253) for 30 min at 4 °C. Cells were pelleted and washed twice in PBS before being resuspended in FACS buffer (PBS, pH 7.4, 5% FBS, 1 mM sodium azide). Cells were then incubated with phycoerythrin (PE)-conjugated anti-human α_5_β_1_ (BD Biosciences, Franklin Lakes, NJ, USA; 555617) or mouse IgG isotype control (BD Biosciences, Franklin Lakes, NJ, USA; 555749) at 1:100 dilution for 30 min. Cells were pelleted by centrifugation, washed twice with cold FACS buffer, and flow cytometric analysis was performed immediately using a BD FACSCanto (Integrated Imaging Center, Johns Hopkins Institute for NanoBioTechnology).

### 2.12. Liposome Internalization in Organoids via Confocal Microscopy

To study liposome internalization in 2D, 5000 cells/well of MDA-MB-231 cells were seeded in a glass-bottom 96-well culture plate. After cells were allowed to adhere overnight, cells were treated with 150 μM Cy5-loaded PR_b liposomes or non-targeted liposomes in media for 24 h at 37 °C. Following treatment, cells were washed with PBS three times and fixed with 4% paraformaldehyde solution for 15 min. Cell nuclei were then stained with Hoechst 33342 at 1:1000 dilution for 10 min and imaged.

To study internalization in 3D, MDA-MB-231 cells were prepared in multi-compartment organoids as described above. Four-day-old organoids were then treated with 300 μM Cy5-loaded PR_b liposomes or non-targeted liposomes in media for 72 h at 37 °C. Following treatment, cells were washed with PBS three times and fixed using 4% paraformaldehyde solution overnight. Cell nuclei were then stained with Hoechst 33342 at 1:250 dilution for 1 h. The organoids were then washed and placed on a glass Petri dish for imaging. Cells were imaged using a Nikon A1 confocal microscope (Nikon Instruments, Melville, NY, USA) and multiple z-scans were collected for every field of view.

### 2.13. Confocal Microscopy of Organoids with Live/Dead Assay

Four-day-old organoids were treated with a live/dead assay that was preformed according to the manufacturer’s protocol. Briefly, cells were exposed to 2 μM calcein-AM and 3 μM propidium iodide for 4 h (Sigma-Aldrich, R37601). Following incubation, the organoids were imaged live using a Nikon A1 confocal microscope.

### 2.14. Cytotoxicity Studies

The IC50 values of free drugs were determined by treating organoids with different concentrations of iMEK or DOX added directly to the culture media for 72 h. Following treatment, cytotoxicity was measured using the PrestoBlue assay compared to an untreated control. The log of drug concentration was then plotted against cell viability, the data fit using the Hill equation in Prism 7, and the IC50 value extracted.

The cytotoxicity of hydrogels encapsulating different therapeutics was assessed in a 96-well transwell plate. Hydrogels containing free drugs, targeted, or non-targeted liposomes were loaded into the upper inserts and maintained at 37 °C for 1 h. After incubating the organoids with hydrogels at 37 °C for 72 h, the media for all treatment groups were exchanged for fresh media. After 24 h, cytotoxicity was measured using the PrestoBlue assay. The same transwell inserts were immediately placed on top of new four-day-old organoids and the experiment was repeated on a total of four batches of organoids over 12 days. Cytotoxicity was calculated as the percentage compared to non-treated controls. Bliss expectation was calculated as (A + B) − (A × B), where A and B are the fractional growth inhibitions induced by agents A and B at a given dose, respectively. Delta Bliss was calculated as the difference between the Bliss expectation and actual observed inhibition of the combination of A and B at a given dose [41]. 

### 2.15. Hydrogel Safety and Biodegradation

To determine the degradation profile and safety of the hydrogels, 5-week-old female *BALB/c* and *NSG* mice (Jackson Laboratories, Bar Harbor, ME, USA) were injected with polymer solution and tracked over time. The hair was removed from the left hind flank of the mice, 200 μL of 22 *w*/*v*% hydrogel solution in PBS was injected subcutaneously, and the volume of the implant was measured via calipers over time. At specific time points, animals were sacrificed and the injection site was excised for macroscopic observation. Samples were also fixed in 10% formalin and sectioned for H&E assessment. The liver, spleen, and kidneys were also harvested for cytotoxicity assessment. The tissue scans were evaluated by a doctor of veterinary medicine (DVM) and an American College of Veterinary Pathologists (ACVP) board-certified pathologist at the Phenotyping Core for pathology support at Johns Hopkins.

### 2.16. Animal Studies

To prepare the human xenograft tumor model, 1 × 10^6^ MDA-MB-231 Ecad+ cells in a 1:1 mix of PBS/Matrigel were injected into the second mammary fat pad of 5-week-old female *NSG* mice (Jackson Laboratories). After the tumor reached ~200 mm^3^, mice were randomly assigned to 6 groups. Mice receiving hydrogel treatments were injected once intratumorally with 50 μL of hydrogel-nanoparticle solution and twice peritumorally with 125 μL (300 μL total) of an aqueous solution containing 22 *w*/*v*% polymer and 9 mM lipids. Controls were injected in the same manner with PBS or empty hydrogel. Hydrogel injections contained liposomes with 1.5 mg/kg iMEK and 3.6 mg/kg DOX per 300 μL and were given every 14 days. 

For intravenous administration of the nanoparticles, a total of 0.75 mg/kg iMEK and 1.8 mg/kg DOX was given via once-weekly injection for five weeks. The tumor volume was calculated from the x,y dimensions measured every three days with calipers and mouse weights were recorded. To mimic standard of care, iMEK was orally administered via peanut butter pellets daily at a dosage of 20 mg/kg for 5 days followed by a 2-day rest period. The treatment cycle was then repeated three times. Tumors were categorized as spheres or ellipses by calculating the difference between x and y. If x − y < 1 mm, the tumor volume was calculated using the volume formula for a sphere. If x − y > 1 mm, the tumor volume was calculated using the volume formula for an ellipse. Tumor-bearing mice were sacrificed when the tumor volume exceeded 1500 mm^3^ or when body weight loss exceeded 20%. Upon sacrifice, mouse organs were harvested and fixed in formalin and sent for sectioning and staining to the Oncology Tissue Services Core at Johns Hopkins. 

For the syngeneic mouse model, 1 × 10^5^ 4T1-luc cells in a 1:1 mix of PBS/Matrigel were injected into the second mammary fat pad of 5-week-old female *BALB/c* mice. After the tumor reached ~200 mm^3^, mice were randomly assigned to 3 groups and injected as in the previous mouse model. Mice receiving the hydrogel treatment were injected once intratumorally with 50 μL of hydrogel-nanoparticle solution and twice peritumorally with 125 μL (300 μL total) of an aqueous solution containing 22 *w*/*v*% polymer and 9 mM lipids. Controls were injected in the same manner with PBS or empty hydrogel. Hydrogel injections contained liposomes with 1.5 mg/kg iMEK and 3.6 mg/kg DOX per 300 μL and were given every 14 days. Tumor burden was observed using the IVIS Spectrum Imaging System, as described below. Tumor-bearing mice were sacrificed when the tumor volume exceeded 1500 mm^3^ or when body weight loss exceeded 20%. Upon sacrifice, mouse organs were harvested and fixed in formalin and sent for sectioning and staining to the Johns Hopkins Oncology Tissue Services Core. 

### 2.17. qPCR of Harvested Mouse Tissue

Mouse lung tissue was broken down with a tissue homogenizer and DNA was extracted from tissue using the PureLink Genomic DNA mini kit (Thermo Fisher, K182002). qPCR was conducted with iTaq-SYBR Green (Bio-Rad, Hercules, CA, USA; 1725121) using the Bio-Rad CFX Touch Real-Time PCR detection system. Primers listed in Table 1 were obtained from Integrated DNA Technologies (Baltimore, MD, USA).

### 2.18. In Vivo Bioluminescence and Imaging

Mice were monitored by bioluminescence imaging for local tumor growth at predetermined time points. Briefly, 10 min after i.p. injection of D-luciferin (GOLDBIO, LUCNA-100) at a dosage of 150 mg/kg, mice were anesthetized with 2% isoflurane and imaged using the IVIS Spectrum Imaging System (Perkin Elmer, Molecular Imaging Service Center, Waltham, MA, USA). Ex vivo images of organs were acquired by sacrificing animals 10 min after i.p. injection. Organs were harvested and submerged in a 300 μg/mL luciferin bath for 5 min before imaging. Bioluminescence images were analyzed using Living Image software 4.5.5 and fluorescence intensity was quantified as the average radiance (photons s^−1^ cm^−2^ sr^−1^).

### 2.19. Immunohistochemistry and H&E Staining of Tissue Sections

Immediately after excision, a portion of the tumor was fixed in formalin and then sent for paraffin embedding and staining to the Oncology Tissue Service Core at JHMI. Tumor slices were stained for proliferation marker Ki67 and apoptotic marker cleaved caspase 3, in addition to H&E for visualization of the tumor microenvironment. 

### 2.20. Tumor Dissociation and Myeloid and T-Cell Subset Analyses

The 4T1 tumors from mice were minced and incubated in RPMI medium containing collagenase/hyaluronidase and DNAse I (Stemcell Technologies, Cambridge, MA, USA) for 30 min at 37 °C. The dissociated tumor was then passed through a 70 µm strainer, pelleted, and resuspended in ammonium chloride solution for 5 min at room temperature. Cells were then washed, counted, and resuspended at 2 × 10^6^ cells in 100 µL of BD Horizon Brilliant Stain Buffer (BD Biosciences) containing 1× Tandem stabilizer (Biolegend, San Diego, CA, USA). FcR on the cells was blocked with TruStain FcX (anti-mouse CD16/CD32) Antibody (Biolegend, San Diego, CA, USA). Cells were then stained with antibodies listed in Table 2. Samples were analyzed using an LSRFortessa Flow Cytometer (BD Biosciences, Franklin Lakes, NJ, USA). Gating was performed on FlowJo 10.9.0 and, subsequently, immune cell subsets were quantified.

### 2.21. Statistical Analysis

Statistical analysis was performed using GraphPad Prism 5. Statistical differences were determined using the unpaired two-tailed Student’s *t*-test or one-way ANOVA was performed with Tukey’s post hoc analysis. Survival was plotted using Kaplan–Meier curves and assessed using two-sided log-rank (Mantel–Cox) tests. 

## 3. Results

### 3.1. E-Cad-Positive Breast Tumors Preferentially Uptake α_5_β_1_ Integrin-Targeted Liposomes and Respond to MEK Inhibition

To overcome the narrow therapeutic index of MEK inhibitors given orally, we employed a fibronectin mimetic peptide, PR_b, to enhance the delivery of liposomes loaded with iMEK. Previous studies have demonstrated that PR_b-functionalized liposomes can selectively bind to human TNBC MDA-MB-231 cells and effectively improve the delivery of chemotherapeutics [17]. PR_b-decorated liposomes have been shown to target the α_5_β_1_ integrin receptor [19,20,22] and have been shown to enhance cellular uptake in pancreatic cancer cells upon release from the thermosensitive and biodegradable PVLA-PEG-PVLA hydrogel in vitro [21]. 

**Figure 1:**
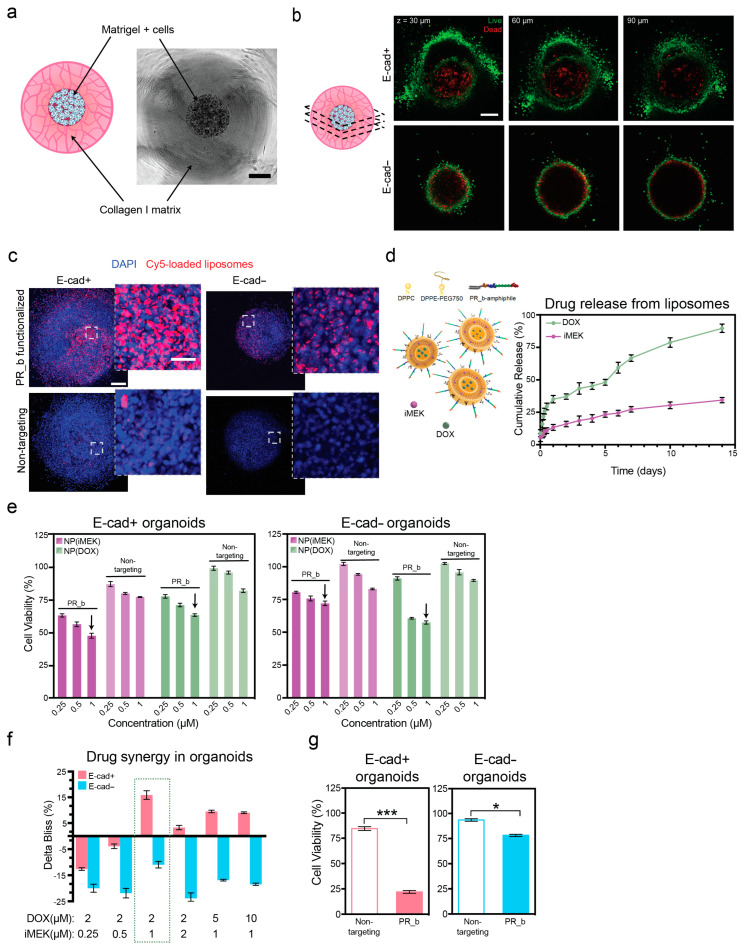
Preferential uptake of targeted liposomes in E-cad-positive MDA-MB-231 cells and increased MEK inhibitor efficacy. (**a**) Schematic of the 3D two-compartment organoid system composed of a Matrigel and cell inner core and collagen I outer layer, with a representative phase-contrast image of the two-compartment organoid system, scale bar is 250 μm. (**b**) Representative confocal laser scanning microscopy z-stack images from live/dead staining of E-cad+ and E-cad− MDA-MB-231 organoids at different cross-sections. Live cells are shown in green and dead cells are shown in red, scale bars are 200 μm. (**c**) Maximum-intensity projection of a confocal microscopy image of Cy5 labeled liposomes (PR_b functionalized and non-targeted) after incubation with MDA-MB-231 organoids for 72 h at 37 °C. Nuclei are shown in blue and liposomes shown in red, scale bar is 200 μm and inset scale bar is 50 μm. (**d**) Schematic of liposomes used in subsequent experiments with release profiles of iMEK and DOX loaded in liposomes at 37 °C. (**e**) Cytotoxicity assessment of iMEK- and DOX-loaded liposomes (both PR_b and non-targeted) at different concentrations on E-cad+ and E-cad− MDA-MB-231 organoids after 72 h. Arrows indicate optimal concentrations used as a starting point for future experiments. Statistical significance was determined via one-way ANOVA with Tukey’s HSD post-hoc analysis and showed that all PR_b formulations were significant (*p* < 0.01) when compared to their non-targeted pair. (**f**) Synergy calculations for the drug interaction between iMEK and DOX (loaded in PR_b liposomes), calculated by plotting cell killing observed in excess of the additive Bliss expectation between iMEK and DOX in E-cad+ and E-cad− MDA-MB-231 organoids. The green dotted box indicates the combination with greatest synergy against E-cad-induced hyper-proliferation in organoids. (**g**) Cytotoxicity assessment of liposomes loaded with 1 μM iMEK + 2 μM DOX delivered with targeted or non-targeted liposomes to MDA-MB-231 organoids after 72 h. Statistical significance was determined using a two-sided unpaired *t*-test; * *p* < 0.05, *** *p* < 0.001.

We began by studying E-cad+ and E-cad− TNBC cell growth progression in a novel multi-compartment tumor organoid system (Figure 1a) [30]. We conducted live/dead staining of cells in the organoids (Figure 1b and Figure S1b), and observed a necrotic core in both E-cad− and E-cad+ organoids on day 7. We determined that the optimal intervention for nanoparticle delivery would be on day 4 of culture, when the proliferation rates (i.e., fold change in PrestoBlue measurements) between E-cad+ and E-cad− organoids were not significantly different, thus ensuring cell cycle changes did not impact uptake (Figure S1c) [42]. With organoid growth behavior and the optimal intervention point determined, we next studied how E-cad manipulation impacted α_5_β_1_ integrin expression in the organoid system by conducting flow cytometry on cells cultured in the organoids (Figure S1d). We observed more than 2-fold increase in the expression of α_5_β_1_ integrin on the surface of MDA-MB-231 E-cad+ organoids compared to MDA-MB-231 E-cad− organoids (Figure S1d). MDA-MB-231 cells do not endogenously express E-cad, so the increase in α_5_β_1_ integrin expression could be correlated with the gain in E-cad expression.

Targeted liposomes were generated with 3.4 ± 0.5 mol% PR_b on the surface, with a size of 126 ± 2.3 nm and a zeta potential of 12.2 ± 0.8 mV. This positive zeta potential developed with the inclusion of the positively charged PR_b peptide-amphiphile, as the non-targeted liposomes were 118 ± 1.8 nm in size with a zeta potential of −14.1 ± 1.1 mV (Table S1). Liposomes visualized with cryogenic transmission electron microscopy (cryo-TEM) showed unilamellar vesicles with the presence of a few multivesicular vesicles (Figure S1e). To functionally demonstrate the preferential binding of PR_b liposomes to E-cad+ organoids, we conducted an uptake experiment with Cy5.5-loaded liposomes (Figure 1c). The increase in α_5_β_1_ integrin expression on the surface of E-cad+ organoids led to increased uptake of the PR_b liposomes when compared to E-cad− organoids (Figure 1c), as anticipated based on the integrin expression results (Figure S1d). We also demonstrated the benefit of adding a targeted peptide to the liposomes, as organoids showed minimal uptake of the non-targeted liposomes after 72 h (Figure 1c). Furthermore, we compared cell association with nanoparticles using flow cytometry and found that TNBC cell monolayers showed 15 times higher nanoparticle signals when compared to monolayers of non-cancerous, healthy breast cell line 184B5 (Figure S1f,g), confirming the preferential uptake of PR_b-functionalized liposomes by cancerous cells. With confirmation that α_5_β_1_ integrin is an effective target for E-cad+ breast cancer organoids and an established treatment timeline, we validated the design of the nanoparticles for the proposed DDS. 

From a clinical perspective, the addition of a toxic inhibitor therapeutic to standard-of-care chemotherapies offered to patients with IDC breast cancer is infeasible, as chemotherapeutics employed for IDC, specifically the TNBC subset, cause significant side effects without the added burden of an MEK inhibitor [43]. When designing our proposed DDS, we wanted to not only lower the off-target toxicity of iMEK PD0325901 but also demonstrate the feasibility of co-delivering iMEK with a traditional chemotherapeutic, i.e., DOX. To co-deliver these two small molecules, we encapsulated hydrophilic DOX (DOX-HCl) inside the liposome, with hydrophobic iMEK contained inside the lipid membrane, achieving 74% encapsulation efficiency (EE) of iMEK and 97% EE of DOX (Figure 1d and Table S2). We then investigated the release of iMEK/DOX from the liposomes and observed a burst release in the first 12 h followed by a slower release, with 34 ± 1% of iMEK and 90 ± 1% of DOX released in 14 days (Figure 1d). Previous studies have reported that the co-delivery of an MEK inhibitor with cisplatin can decrease cell viability in melanoma and colon cancer, but these studies were conducted in 2D models, lacking physiological relevance [10,11]. To demonstrate the importance of the PR_b peptide, targeted and non-targeted liposomes were delivered to E-cad+ and E-cad− organoids for 72 h, after which organoid viability was assessed. As anticipated, based on the nanoparticle uptake data, the incorporation of PR_b significantly improved the efficacy of both iMEK- and DOX-loaded liposomes (Figure 1e). While DOX-loaded liposomes reduced viability similarly between E-cad+ and Ecad− organoids, iMEK-loaded liposomes were much more effective against E-cad+ organoids, as expected from the observed IC50 of 0.38 μM for E-cad+ cells and 1.7 μM for E-cad− cells (Figure S1h). 

Additionally, we investigated the potential synergy of co-delivery treatment between iMEK and DOX using targeted liposomes, as measured by the amount of synergistic killing in excess of the additive Bliss expectation [41]. The Bliss model of synergy was chosen because of the independent mechanisms of action by iMEK and DOX. The drug combination was synergistic in E-cad+ organoids when the iMEK concentration was at or above 1 μM for different DOX concentrations, while the combination did not demonstrate synergy in E-cad− organoids (Figure 1f). This result agreed with previous findings, as without the hyper-active MEK/ERK cascade, iMEK has little impact on cell viability and therefore cannot be expected to produce a synergistic killing effect with a chemotherapeutic [7]. The combination of 1 μM iMEK with 2 μM DOX dramatically reduced cell viability in E-cad+ organoids by more than 75% (Figure 1g) compared to either drug alone (Figure 1e). This drug combination was not very effective at reducing viability in E-cad− organoids, as anticipated. Furthermore, PR_b liposomes were still better at killing organoids than non-targeted liposomes when loaded with iMEK/DOX, demonstrating the need for targeting. Thus, 1 μM iMEK with 2 μM DOX was selected for further in vitro studies, as it decreased cell viability to similar levels when compared with higher drug concentrations (Figure S2a). 

### 3.2. PVLA-PEG-PVLA Hydrogel Is Thermosensitive and Provides Sustained Release of Targeted Nanoparticles Encapsulating iMEK and DOX

**Figure 2:**
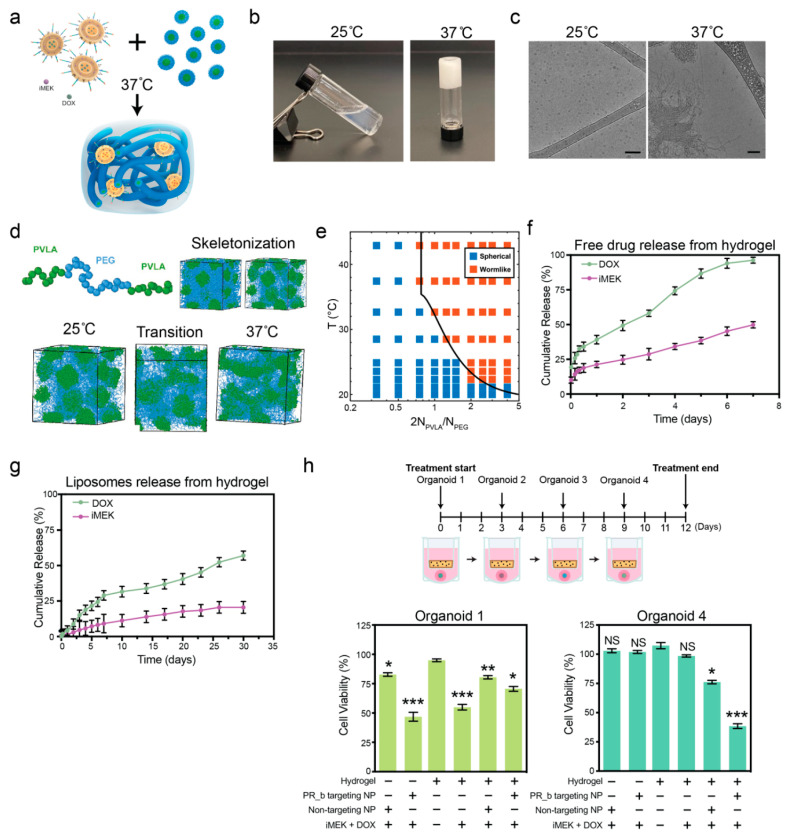
Hydrogel characterization and extended-release evaluation. (**a**) Schematic illustration of the PVLA-PEG-PVLA hydrogel with liposomes, demonstrating the polymer phase transition and entrapment of liposomes in the hydrogel. (**b**) PVLA-PEG-PVLA polymer as a liquid at 25 °C and a gel at 37 °C as confirmed by tube inversion. (**c**) Cryo-TEM images of PVLA-PEG-PVLA solution in water (0.25% *w*/*v*) at 25 °C and 37 °C, scale bar is 100 nm. (**d**) Coarse-grain molecular simulation of the spherical-to-wormlike micelle transition of PVLA-PEG-PVLA upon heating. Top right image shows a snapshot with the full PEG block (**left**) and skeleton (**right**). Bottom images show simulation snapshots of the self-assembled micelle structure below, near, and above the spherical-to-wormlike transition temperature for $2N_{PVLA}/N_{PEG}=0.75$. Visualization shows the PEG block (blue) in skeletal lines to better highlight the individual structures. (**e**) Composite phase diagram of temperature versus $2N_{PVLA}/N_{PEG}$ ratio at 22 *w*/*v*%. Scatter points indicate simulation results. The solid black line is theory prediction from Equation (8). The vertical line indicates the asymptote of theory prediction, below which all triblock copolymers form spherical micelles. (**f**) Release profiles of iMEK and DOX loaded as free drugs entrapped in the hydrogel or (**g**) in liposomes in the hydrogel at 37 °C. All results are reported as the mean ± SEM (*n* = 3). (**h**) Schematic of a 12-day organoid growth inhibition experiment with MDA-MB-231 4-day-old organoids (i.e., at t = 0, organoids are at t_growth = 4 days) exposed to different samples via a transwell insert. After each 3-day treatment, the same transwell inserts were employed for new 4-day-old organoids. Cell viability of MDA-MB-231 organoids compared to non-treated controls. Data are the mean ± SEM (*n* = 3). Statistical significance determined via one-way ANOVA with Tukey’s HSD post hoc analysis and *p*-values shown in comparison to the empty hydrogel control; * *p* < 0.05, ** *p* < 0.01, *** *p* < 0.001, NS: not significant.

The PVLA-PEG-PVLA triblock copolymer was synthesized as previously reported [27]. The number average molecular weight was confirmed by ^1^H NMR to be 1570-1500-1570. The triblock copolymer underwent a liquid–gel transition via a spherical-to-wormlike micelle transformation (Figure 2a). The PVLA-PEG-PVLA triblock copolymer formed a liquid at room temperature and an opaque hydrogel upon heating to physiological temperature (Figure 2b). Cryo-TEM was used in the dilute limit (0.25 *w*/*v*%) to confirm the transition from spherical to cylindrical micelles, the highly clustered nature of which suggested some amount of triblock copolymer bridging (Figure 2c). This result agreed with previous studies that showed the spherical-to-wormlike transition with cryo-TEM as well as small angle neutron scattering (SANS) measurements at higher concentrations (20 *w*/*v*%) [21,27]. This phase transition is uncommon and has been shown before in triblock copolymers, such as certain pluronics, at high temperatures (T ≥ 60 °C) or in the presence of high salt concentrations [28,29].

To probe the driving forces governing self-assembly of the PVLA-PEG-PVLA triblock copolymer, we developed a coarse-grained model to capture the spherical-to-wormlike micelle transition using molecular dynamics (MD) simulations. Briefly, we used a bead-spring model for the BAB chains, with hydrophobic B and hydrophilic A segments (Figure 2d). We employed a FENE potential [44] for bonded particles and an implicit solvent model for non-bonded interactions, where water (solvent) interactions with PVLA and PEG segments were mediated by an effective pairwise potential between all monomers. Characterization of the self-assembled morphologies revealed the formation of spherical micelles at low temperatures and PVLA/PEG ratios. Increasing either temperature or the PVLA/PEG ratio drove a transition from spherical to wormlike micelles, with coexistence of the two structures at intermediate values (Figure 2d). 

Phenomenologically, the temperature-dependent spherical-to-wormlike transition was a direct consequence of PEG dehydration that occurred at higher temperatures. Initially, PEG-dehydration shrinks the PEG block, making the PEG corona around the PVLA core patchier due to PEG–PEG aggregation and exposing the previously buried PVLA monomers at the center of each micelle to the surrounding water. In other words, increasing temperature results in the emergence of surface patchy micelles in solution that favor aggregation to minimize PVLA–water interactions. However, the presence of the PEG corona around the PVLA core poses steric restrictions that inhibit full micellar merging. Rather, PEG blocks get “pushed” away from regions with more exposed PVLA monomers to maximize PVLA–PVLA contacts. This mode of patchy-driven aggregation is known to give rise to cylindrical and network formation [45,46,47] and corroborated the spherical-to-wormlike transition observed in experimental settings. We leveraged this phenomenological picture to develop a scaling theory for predicting PVLA-PEG-PVLA triblock copolymer self-assembly. Our theory considered the picture of a spherical core of size $R_{PVLA}$ formed by the aggregated PVLA monomers with the PEG block forming a corona around the core surface, analogous to a “grafted” layer. A temperature rise drives PVLA aggregation and increases core packing, reminiscent of a polymer melt to poor solvent transition. We then defined the size of the PVLA block ($R_{PVLA}$) and PEG block ($R_{PEG}$) and computed the Flory energy of the individual PVLA ($F_{PVLA}$) and PEG ($F_{PEG}$) blocks within the micelle (see Section 2.5). When $F_{PVLA}\;\sim \;F_{PEG}$, PVLA aggregation dominated over steric repulsion due to crowding between neighboring PEG chains. This aggregation provided the driving force to “push” PEG chains away and maximize PVLA–PVLA contact, facilitating the spherical-to-wormlike micelle transition. We predicted the equilibrium morphology as a function of temperature and ratio of PVLA/PEG ($N_{PVLA}/N_{PEG}$) chain lengths in terms of repeat units. Overlaying theory prediction, given by Equation (8), with data from simulations indicated that we could accurately predict the micelle morphological transition across the entire phase space (Figure 2e). The phase diagram closely matched the experiments showing that, at 37 °C, a 22 *w*/*v*% polymer should form a gel. 

Finally, to obtain a more quantitative comparison, we computed the average ratio of the spherical micelle diameter to the diameter of the wormlike micelles from simulations, yielding a value of 1.21 ± 0.09. Analysis of the cryo-TEM images showed a change from 15.4 ± 1.1 nm for the spheres to 13.0 ± 1.0 nm for the fibers, corresponding to a ratio of 1.18, which was in good agreement with our computational findings and previous reports [27]. We additionally performed theory prediction of micellar diameter by assuming a random and densest spherical packing of $N_{PVLA}$ Kuhn monomers in the spherical and wormlike configurations, respectively (see Section 2.5). This prediction yielded a theoretical ratio of 1.18, in excellent agreement with both experimental and simulation results. Our proposed theory not only elucidates a fundamental understanding of the underlying physics governing micelle morphology but also provides a powerful, predictive tool for subsequent design of materials utilizing this class of PVLA-PEG-PVLA triblock copolymer.

With the polymer system characterized, we sought to optimize our iMEK- and DOX-loaded, PR_b-functionalized liposomes by testing them with the organoid model and confirming the ability of the hydrogel-nanoparticle system to provide an extended-release platform. To characterize our proposed DDS, we performed fundamental in vitro release studies analyzing the release of drugs into PBS. Based on previous work in which we used the thermosensitive hydrogel to deliver standard-of-care chemotherapeutics to pancreatic cancer spheroids in vitro, the platform should provide extended release [21]. We previously confirmed the ability of the hydrogel to entrap targeted liposomes and that the inclusion of liposomes did not impact the degradation or stiffness of the hydrogel [21]. The hydrogel released 50 ± 2% of iMEK and 96 ± 2% of DOX after only 7 days (Figure 2f). By contrast, the encapsulation of liposomes in the hydrogel resulted in sustained release with 21 ± 4% of iMEK and 57 ± 3% of DOX released after 30 days (Figure 2g). Once the nanoparticles were internalized by cells, the liposomes would release the entire drug payload, allowing for the synergistic cytotoxic effect of iMEK and DOX [48,49]. Thus, encapsulating the liposomes in the hydrogel extended the release timeline of the drugs, providing an optimal platform for sustained release after local delivery. To functionally test the sustained delivery of the liposomes from the thermosensitive hydrogel, we subjected multiple batches of organoids to the same empty hydrogel, hydrogel containing free drugs, or liposomes. 

Over a period of 12 days, four separate batches of organoids were treated on day 4 of organoid culture with the same formulation/DDS (Figure 2h and Figure S2b,c). We tested our proposed system: PR_b liposomes encapsulating both drugs entrapped in the thermosensitive hydrogel against various controls: (1) free non-targeted liposomes encapsulating both drugs, (2) free PR_b liposomes encapsulating both drugs, (3) empty hydrogel, (4) hydrogel containing free drugs, and (5) non-targeted liposomes encapsulating both drugs, entrapped in the hydrogel. As the multi-compartment organoids utilized in these experiments were truly 3D (i.e., floating in medium during culture), we used a transwell insert to treat the organoids with the various treatment groups, allowing for diffusion of the nanoparticles or drugs into the culture medium. Four-day-old organoids were exposed to the different formulations through the transwell inserts for 3 days. At the end of treatment, the organoids were transferred to fresh control medium, and the transwell inserts were immediately transferred to a new batch of untreated 4-day-old organoids. The treated organoids were cultured for 1 more day before the final PrestoBlue viability measurement. 

In the first batch of organoids, both free PR_b liposomes loaded with the two drugs (iMEK/DOX) and hydrogels encapsulating free drugs resulted in reduced organoid viability (Figure 2h). This strong initial effect was markedly different from the organoid viability results of the fourth batch of organoids, in which the PR_b liposomes in the hydrogel had the greatest impact, reducing viability to 38%, compared to the free PR_b liposomes or free drugs in the hydrogel, which did not lower cell viability in a significant manner. The free drugs in the hydrogel had minimal impact on cell viability of the fourth organoid, as 96% of DOX and 50% of iMEK had been released by day 7 (Figure 2f), leaving low amounts of drugs to affect the final organoid. Similarly, the free liposomes had little impact on cell viability since 80% of DOX and 30% of iMEK were already released by day 14 (Figure 1d) from the transwell insert prior to being moved to the fourth organoid. The PR_b liposomes in the hydrogel reduced viability more than the non-targeted liposomes in the hydrogel, demonstrating the benefit of including a cell-targeting ligand and enhancing the cell uptake of liposomes. The results of this study highlight the benefit of using targeted liposomes entrapped in a thermosensitive hydrogel as a delivery system.

### 3.3. PVLA-PEG-PVLA Hydrogel Is Thermosensitive and Biocompatible In Vivo

**Figure 3:**
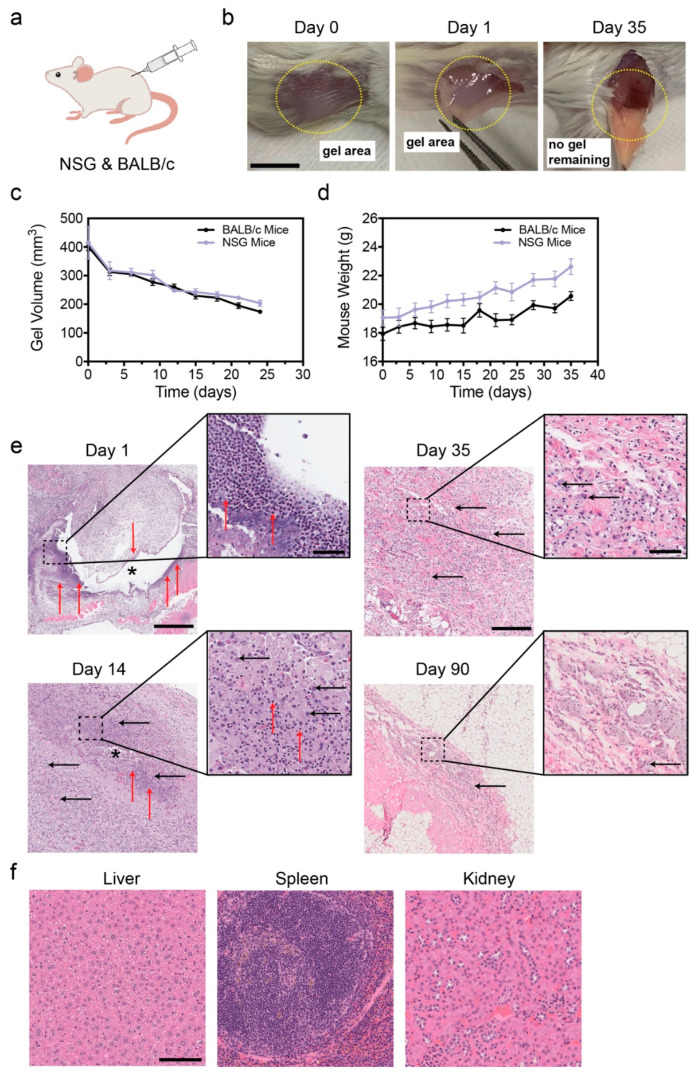
In vivo biodegradation of PVLA-PEG-PVLA hydrogel in the absence of nanoparticles. (**a**) Schematic of subcutaneous hydrogel injection in both *NSG* and *BALB/c* mice. (**b**) In vivo gel formation and retention analyzed after subcutaneous injection of PVLA-PEG-PVLA solution in the flank of *BALB/c* mice, scale bar is 1 cm. (**c**) Degradation rate of hydrogel in *NSG* and *BALB/c* mice as measured by hydrogel volume via calipers. Data are shown as the mean ± SEM (*n* = 4 per group). (**d**) Body weight of *NSG* and *BALB/c* mice after polymer injection. Data are shown as the mean ± SEM (*n* = 4 per group). (**e**) Representative images of H&E staining of injection area at different time points in *BALB/c* mice. Asterisk denotes injection site, vertical red arrows indicate polymorphonuclear neutrophils, and horizontal black arrows indicate foamy macrophages. Scale bar is 500 μm and inset scale bar is 50 μm. (**f**) Representative images of H&E staining of filtration organs from *BALB/c* mice 90 days after polymer injection, scale bar is 100 μm.

To further characterize this unique thermosensitive polymer, we determined the in vivo biocompatibility and biodegradation rate of the hydrogel. To quantify the rate of biodegradation, we conducted in vivo studies in both *NSG* and *BABL/c* mice, as these were the two species of mice used in our later orthotropic mouse models. We subcutaneously injected the polymer into the flank of the animal (Figure 3a) and used calipers to measure the x,y dimensions, allowing us to calculate the volume of the implant. Immediately after injection, a dome was observed under the skin that could be palpated, and no gel leaked from the injection site, confirming the rapid formation of a hydrogel upon reaching physiological temperature. One day after injection, gross macroscopic examination verified gel formation, which was observed as a slightly opaque hydrogel upon dissection (Figure 3b). We also tracked the implant volume degradation over the course of 25 days (Figure 3c), at which point the implant was too small to accurately measure (<150 mm^3^). The biodegradation in vivo study was terminated on day 35, when no implant was palpable, indicating complete degradation. Absence of hydrogel was confirmed by macroscopic observation during dissections (Figure 3b). Throughout the study, mice weights were recorded when the gel implant was measured as a first indication of potential toxicity from the hydrogel formation. We did not observe any weight loss in the animals for the duration of the study (Figure 3d), instead observing steady weight gain as anticipated in healthy animals. 

To capture the various stages of degradation, we sacrificed animals at multiple time points and harvested the gel/site of implantation as well as the liver, kidneys, and spleen. All tissues were fixed and stained with hematoxylin and eosin (H&E), allowing for pathological assessment of biocompatibility One day after the polymer injection, we observed acute inflammation that began resolving by day 14, characterized by the presence of polymorphonuclear neutrophils and foamy macrophages (Figure 3e). On day 35, the inflammation had continually resolved into the formation of fibrotic tissue with few foamy macrophages remaining. We then wanted to verify the long-term impact of the hydrogel and conducted a 90-day study to ensure no permanent changes to the tissue. After three months, the inflammation completely resolved, with few immune cells present and the remaining fibrotic tissue shrinking. Over the course of 90 days, histological analysis of tissues revealed no significant changes in the kidneys, liver, or spleen, indicating no systemic toxicity from the hydrogel (Figure 3f). These results indicated the biodegradable nature of the hydrogel, with no collagenous fibrous capsule forming, chronic inflammation resolving, and no pathological anomalies observed in the reticuloendothelial system (RES) organs when compared to animals that did not receive a polymer injection (Figure S3), demonstrating that the gel was biocompatible. Taken together, these results demonstrate that the hydrogel is biocompatible and suitable for use as a carrier for liposomes in vivo.

### 3.4. Sustained, Local, and Targeted Delivery of iMEK and DOX Effectively Slows E-Cad+ Tumor Progression in a Xenograft Model

**Figure 4:**
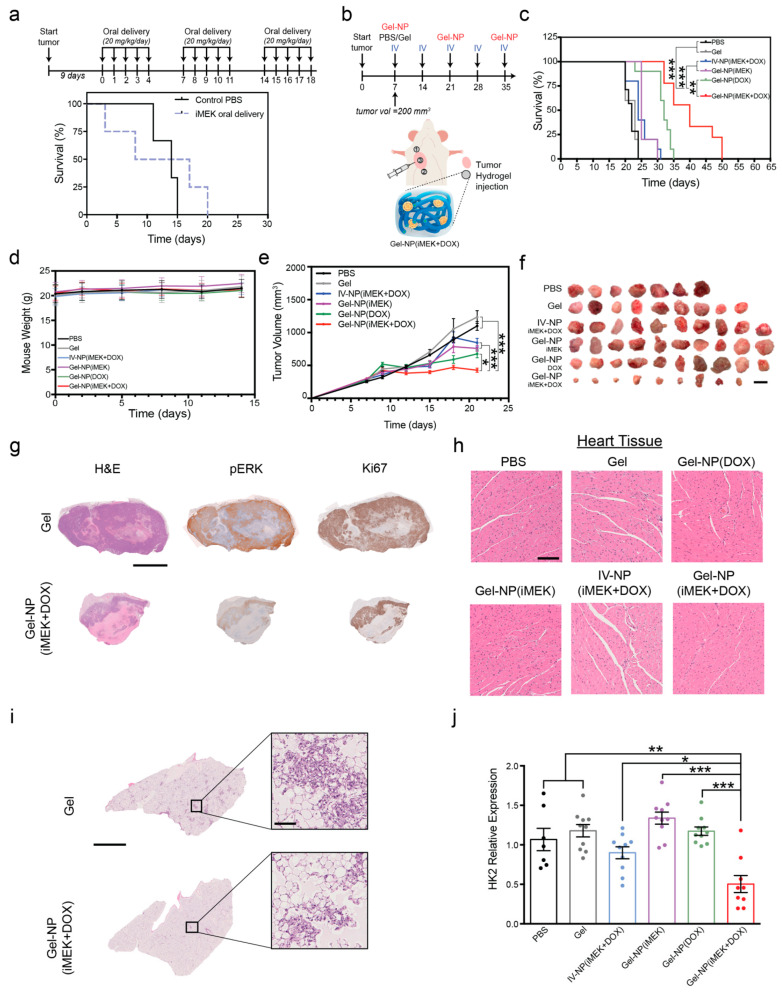
Local and targeted delivery of iMEK and DOX in liposomes in TNBC xenograft mouse model. (**a**) Treatment schedule and survival curve for mice treated with orally delivered iMEK. E-cad+ MDA-MB-231 cells were implanted in the second mammary fat pad of *NSG* mice 9 days before treatment began on day 0. Oral iMEK was given every day for 5 days, followed by 2 days of rest, and the cycle was repeated 3 times. Statistical significance was determined using a two-sided log-rank (Mantel–Cox) test (*n* = 3). (**b**) Preparation and treatment schedule of mice. E-cad+ MDA-MB-231 cells were implanted in the second mammary fat pad of *NSG* mice day 0, 7 days before treatment began. IV nanoparticle injections were given every 7 days and hydrogel injections once every 14 days. Gels were administered via 3 separate injections every 14 days. (**c**) Survival curves corresponding to different treatment groups (*n* = 7–10). Statistical significance was determined using a two-sided log-rank (Mantel–Cox) test. (**d**) Body weight of mice in different groups during treatment. (**e**) Tumor growth in different treatment groups over time. Data are mean ± SEM and statistical significance was determined via one-way ANOVA with Tukey’s HSD post-hoc analysis. (**f**) Scaled pictures of the excised orthotopic tumors from all treatment groups. Scale bar is 10 mm. (**g**) Representative images of H&E-stained tumors and IHC staining for pERK and Ki67. Scale bar is 3 mm. (**h**) Representative images of H&E-stained hearts for various treatment groups, scale bar is 100 μm. (**i**) Representative images of H&E-stained lung sections. Scale bar is 3 mm and inset scale bar is 100 μm. (**j**) Relative expression level of *HK2* in the lungs of treatment groups. Results are presented as the mean ± SEM (*n* = 7–10) and statistical significance determined via one-way ANOVA with Tukey’s HSD post hoc analysis. For all plots in this figure; * *p* < 0.05, ** *p* < 0.01, *** *p* < 0.001.

With confirmation that our polymer-nanoparticle system provided sustained co-delivery of iMEK and DOX encapsulated in targeted liposomes, we sought to test the translatability of our system to animal models. A pre-clinical model of breast cancer would allow us to determine if local, targeted delivery can increase the therapeutic index of iMEK and provide an effective method to translate iMEK combination therapy to the clinical setting. To evaluate and compare our DDS to other methods of delivery, we designed an orthotopic xenograft study of MDA-MB-231 E-cad+ breast cancer in *NSG* mice. To compare with the clinically standard administration route of iMEK, we first ran a pilot study in which iMEK was delivered orally following a dosage schedule that mirrored a clinical trial: animals were orally administered iMEK for 5 days, followed by 2 days of rest, and then the cycle was repeated (Figure 4a). Animal weights were tracked throughout the study, and significant weight loss was seen in the oral delivery cohort (Figure S4a). The systemic delivery of iMEK via oral administration resulted in many off-target and toxic effects, resulting in the deaths of animals before they succumbed to the tumor (Figure 4a), further highlighting the need for local, targeted delivery to overcome this toxicity limitation. 

To deliver the hydrogel-nanoparticle system to mice bearing orthotopic tumors, the thermosensitive polymer solution was mixed with PR_b liposomes containing iMEK and DOX. The polymer-nanoparticle solution was injected directly into the tumor, followed by peripheral tumor injections, a scheme developed based on past work demonstrating that local hydrogel injections can successfully treat breast tumors (Figure 4b) [26,50,51]. Furthermore, any nanoparticles that drained from the tumor site would still be able to target the tumor from systemic circulation due to the targeted peptide [20,22,52]. We compared our novel DDS (Gel-NP(iMEK+DOX)) to the following controls: (1) phosphate-buffered saline injection (PBS), (2) empty hydrogel (Gel), (3) intravenous injection of targeted nanoparticles (IV-NP(iMEK+DOX)), and (4,5) single-agent targeted nanoparticles in the hydrogel (Gel-NP(iMEK) and Gel-NP(DOX)). Compared to the controls (PBS and Gel), the Gel-NP(iMEK+DOX) system doubled both the lifetime of the mice from 24 days to 50 days and the median survival time from 22 days to 40 days (Figure 4c). Mice weights were tracked for the duration of the study to capture any potential toxic effects of the treatments. No significant weight loss was observed in this study, indicating that off-target effects were mitigated (Figure 4d). When assessing the impact of Gel-NP(iMEK+DOX) on tumor volume progression over time, a significant slowing, and in some cases halting, of primary tumor growth was observed compared to all other groups (Figure 4e,f).

At the conclusion of the study, tumors were excised and preserved for further analysis, as well as organs of interest (i.e., lungs, liver, kidneys, spleen, pancreas, and heart). The final tumor weight confirmed the effectiveness of the treatments on tumor proliferation and growth. When comparing the Gel-NP(iMEK+DOX) group’s final tumor weights to those of all other groups, a significant reduction in tumor weight of 56% compared to the PBS control was observed (Figure S4b). We also performed H&E and immunohistochemistry (IHC) analysis on the excised tumors and observed a lowering of ERK phosphorylation (pERK) and subsequently Ki67, as expected given the proposed mechanism of iMEK inhibition [7] (Figure 4g and Figure S4c). Histological examination of heart tissues, the most common place for side effects from DOX therapy, showed no abnormal tissue changes in any group, except a slight decrease in muscle cell size in the IV group (Figure 4h) [53,54]. Similarly, there were no significant findings in the liver, spleen, or kidneys of any group (Figure S4d), indicating that all treatments were effective in limiting the systemic toxicity associated with delivery of free DOX, such as nephrotoxicity or hepatotoxicity [55]. 

We further investigated if local delivery of Gel-NP(iMEK+DOX) had an impact on tumor metastasis. Significant reduction in lung metastases, the most common metastatic site in TNBC, was evident from H&E sections of lungs that were inflated with agarose and then fixed in formalin after excision (Figure 4i and Figure S4e) [56,57]. We also performed quantitative polymerase chain reaction (qPCR) analysis on lungs excised from mice and measured the expression of human genomic material marker, *HK2*. The Gel-NP(iMEK+DOX) group exhibited a significant reduction in metastatic burden when compared to all other experimental groups (Figure 4j). These results showed that the locally delivered hydrogel-nanoparticle system could reduce tumor burden at the primary site as well as metastatic burden.

### 3.5. Sustained, Local Delivery of iMEK and DOX Effectively Slows Tumor Progression and Metastasis in a Syngeneic Mouse Model of TNBC

**Figure 5:**
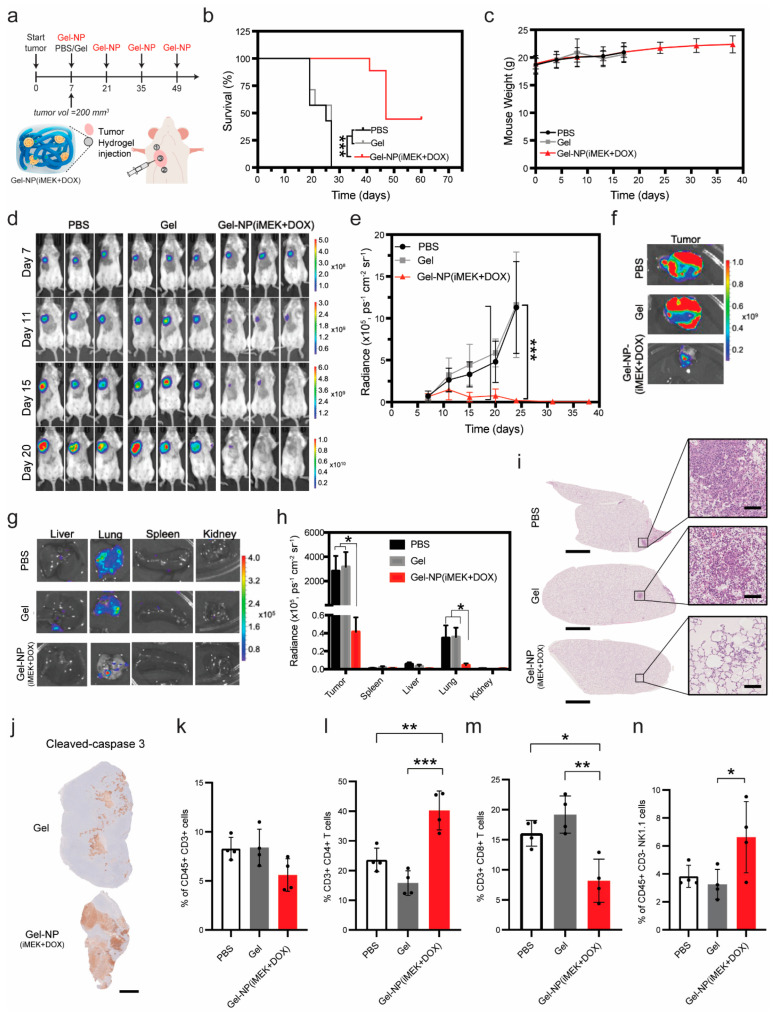
Local and targeted delivery of iMEK and DOX in liposomes in syngeneic mouse model. (**a**) Preparation and treatment schedule of mice. 4T1-luc cells were implanted in the second mammary fat pad of *BALB/c* mice on day 0, 7 days before treatment began. Hydrogels were injected every 14 days. Gels were administered via 3 separate injections on each day of treatment. (**b**) Survival curves corresponding to different treatment groups (*n* = 7–9). Statistical significance was determined using a two-sided log-rank (Mantel–Cox) test. (**c**) Body weights of mice in different groups during treatment. (**d**) Representative bioluminescence images of mice at different time points. (**e**) Quantification of tumor bioluminescence values in different treatment groups over time. Data are shown as means ± SEM. Statistical significance on day 17 was determined via one-way ANOVA with Tukey’s HSD post-hoc analysis. (**f**) Bioluminescence images of excised orthotopic tumors upon animal death. (**g**) Bioluminescence images of excised organs upon animal death. (**h**) Quantification of excised tumor and organ bioluminescence values at the end of study. Values are shown as the mean ± SEM. Statistical significance between organs of different groups was determined via one-way ANOVA with Tukey’s HSD post hoc analysis. (**i**) Representative images of H&E-stained lung sections. Scale bars for full sections are 3 mm and those of high-magnification insets are 100 μm. (**j**) Representative images for IHC staining of apoptosis marker cleaved caspase 3, scale bar is 3 mm. (**k**–**n**) Flow cytometric quantification of immune cells from 4T1 tumors, (**k**) all T cells (CD3+), (**l**) CD4+ T cells, (**m**) CD8+ T cells, and (**n**) NK cells. Statistical significance was determined via one-way ANOVA with Tukey’s HSD post hoc analysis. For all plots in this figure; * *p* < 0.05, ** *p* < 0.01, *** *p* < 0.001. For all unbracketed groups, *p* > 0.05.

To further demonstrate the translation potential, we tested our hydrogel-nanoparticle system in a syngeneic mouse model of breast cancer, which also allowed us to study how the immune system responded to our treatment. Murine breast cancer cell line 4T1 was injected in the mammary fat pad and grown for 1 week to initiate orthotopic breast tumors [38]. Next, we injected the polymer-nanoparticle solution directly into the tumor (site 1, Figure 5a) and the tumoral periphery (sites 2 and 3, Figure 5a). We utilized a luciferase-tagged 4T1 cell line (4T1-luc), allowing us to monitor tumor volume as a function of radiance via the IVIS Imaging System, as well as caliper measurements. The luciferase tag also allowed us to assess metastatic spread at the conclusion of the study. 

We compared our novel hydrogel-nanoparticle DDS Gel-NP(iMEK+DOX) to PBS and empty gel controls. The Gel-NP(iMEK+DOX) treatment increased median survival by a factor of approximately 2, similarly to how it performed in the *NSG* mouse model. No difference was observed between the control groups (PBS and empty hydrogel injections) either, aligning with observations from the *NSG* study (Figure 5b). Furthermore, Gel-NP(iMEK+DOX) resulted in long-term survival due to a halt in tumor progression for 4 out of 9 mice. Importantly, mouse weight was tracked for the duration of the study to capture any potential toxic effects of the treatments, and no significant animal weight loss was observed in this study (Figure 5c). This was supported by analysis of the H&E sections of the liver, spleen, heart, and kidneys that were collected upon termination of the study, which showed no pathological abnormalities in response to the treatment (Figure S5d). The Gel-NP(iMEK+DOX) treatment resulted in a nearly complete loss of radiance in the tumor mass by day 22 of the study, suggesting eradication of tumor cells (Figure 5d,e). We also observed a significant decrease in tumor volumetric progression in the treatment group, as calculated from the x,y dimension caliper measurements (Figure S5a), confirming what was observed in the *NSG* survival study. Images of excised tumors further confirmed that the hydrogel-nanoparticle system killed most of the tumor cells, as treated tumors displayed only a small area of luciferase-positive cells at the conclusion of the study (Figure 5f and Figure S5b). H&E staining of tumor sections revealed that by the end of the study, treated tumors consisted of collagen and other extracellular matrix components, with much fewer cancer cells present compared to the PBS and empty hydrogel controls (Figure S5c). Together, these data indicated that our proposed hydrogel-nanoparticle system, designed to deliver iMEK+DOX locally and slowly, could slow down and even halt tumor progression.

To assess the impact of our treatment on metastasis, we performed both IVIS imaging and analyzed H&E sections of excised livers, lungs, spleens, and kidneys at the end of the study. The Gel-NP(iMEK+DOX) treatment group had significantly decreased luciferase signals in the lungs when compared to the control groups (Figure 5g,h). There were no visible areas of luciferase signal in the livers of the Gel-NP(iMEK+DOX) group (Figure 5g), but the difference was not significant when compared to both control groups (Figure 5h).

No major metastasis was present in H&E sections of the liver (Figure S5d). No group showed positive signals in the spleen or kidneys (Figure 5g,h and Figure S5d). We further confirmed the absence of cancer cells in the lungs of the GEL-NP(iMEK+DOX) group via H&E staining of lung sections. There were macro-metastases in both control groups (PBS and empty gel) but not in the Gel-NP(iMEK+DOX) group, showing that our treatment minimized metastasis (Figure 5i). H&E sections from kidneys, livers, hearts, and spleens of the PBS control group, empty gel (Gel) group, and Gel-NP(iMEK+DOX) treatment group did not contain any cancer cells (Figure S5d). The low levels of metastasis observed in the treatment group were likely the result of cancer cells that disseminated from the primary tumor in the first week of growth, prior to injection of the hydrogel. 4T1 cells are particularly aggressive and previous studies have shown that 4T1 cells quickly metastasize, and once the cancer cells infiltrate the lung tissue, treatment has little to no impact on preventing metastatic growth [58,59]. Despite similar levels of spleen white pulp between all groups (Figure S5d), the Gel-NP(iMEK+DOX) system suppressed the enlargement of spleens by approximately 50%, as measured by weight (Figure S5e). Splenomegaly is a sign of myeloid cell expansion due to inflammation caused by cancer, which can lead to major issues in blood cell count and health [60,61,62]. 

To further explore the tumor microenvironment, we performed H&E and IHC staining on tumor sections. H&E staining revealed that the dual-therapy hydrogel-nanoparticle system reduced the density of cancer cells in the tumor microenvironment, marked by the lack of nuclei in the tumor masses that were excised (Figure S5c). We also stained for apoptotic marker cleaved caspase 3 and proliferation marker Ki67. We observed an increase in cleaved caspase 3 signal in the Gel-NP(iMEK+DOX) group compared to both PBS and empty gel control groups (Figure 5j and Figure S5g). All tumors showed a necrotic core with a population of apoptotic cells and lack of cells in the core, as evident in H&E and IHC staining (Figure S5c,f), confirming our observations in the organoid model (Figure 1b). We also observed a decrease in the Ki67 signal in our dual-therapy hydrogel-nanoparticle group (Figure S5f). A decrease in proliferative cells was expected given the cytotoxic nature of the therapies we delivered to the tumor. 

To assess the interplay of the immune response and the Gel-NP(iMEK+DOX) system and empty gel (Gel), we performed flow cytometry on tumors from all groups. In an attempt to understand how the co-delivery of DOX and iMEK resulted in long-term survivors, we chose to quantify the following immune cell populations: macrophages, neutrophils, dendritic cells, T cells, B cells, and natural killer (NK) cells (Figure S6). We observed a significant increase in the CD4+ T cell population in the Gel-NP(iMEK+DOX) group compared to both the PBS and empty gel groups (Figure 5l), while there was no significant difference in the total T cells between groups (Figure 5k). This increase in T-helper cell (CD4+) population without an increase in total T cells was quite intriguing, indicating a shift in phenotype without an increase in overall production or requirement of T cells to the tumor microenvironment. Conversely, the CD8+ population was lower in our combination treatment compared to the controls (Figure 5m). We also observed an increase in the NK cell population in the Gel-NP(iMEK+DOX) group when compared to the empty gel group (Figure 5n). All other immune cells populations analyzed did not exhibit statistically significant differences between the different groups (Figure S7). In summary, our novel hydrogel-nanoparticle system can be utilized to both slow tumor progression and reduce metastatic burden, with an overall positive impact on survival rate.

## 4. Discussion 

Sustained, targeted, and local delivery can be utilized to overcome the toxicity limitations of MEK inhibitors in the treatment of breast cancer. This study demonstrates that targeted liposomes encapsulating iMEK and DOX can be delivered via a thermosensitive and biodegradable hydrogel to stabilize tumor progression and decrease metastasis in E-cad+ TNBC breast tumors. Currently, the combination of chemotherapeutics with MEK inhibitors is under clinical investigation, with extensive studies completed in non-small cell lung cancer and some trials in TNBC [63,64]. Tumors extracted from patients resistant to conventional chemotherapeutics, such as DOX or docetaxel, were found to have elevated RAS-RAF-MEK-ERK activation, making the addition of MEK inhibitors to downregulate this pathway a promising approach for the resistant TNBC subtype of IDC [65]. The combination of the MEK inhibitor, cobemetinib, plus paclitaxel led to an increase in progression-free survival and objective response rate, although the results were not significant [64]. Though promising, the study required frequent dosing of the drugs and saw adverse events such as diarrhea, highlighting the need for a targeted, extended-release system that can be locally delivered, thus avoiding the need for systemic delivery of widely acting MEK inhibitors. There has been one preclinical evaluation of an MEK inhibitor delivered through liposomes locally; however, the nanoparticles were injected intratumorally without a hydrogel scaffold to provide extended release. This therapy required frequent injections every other day and the efficacy dropped as soon as regular injections stopped [66]. 

Here, we provide evidence that targeted, sustained, local delivery can mitigate the adverse effects associated with systemic delivery. Our system allows for the co-delivery of two anti-proliferative treatments that act synergistically and directly at the tumor site without evidence of severe toxicity in two animal models, due to the targeted component of the liposomes and localized delivery of the unique thermosensitive hydrogel. Furthermore, our system allows for fewer therapeutic administrations than the clinical gold standard of I.V. nanoparticle administration.

In this study, we proposed to utilize E-cad as a biomarker, indicating that the breast tumor would respond well to MEK inhibition, based on our recent study in which E-cad expression resulted in hyper-activation of ERK, downstream of MEK [7]. E-cad expression data are routinely collected as they are used to help determine if a breast tumor is a ductal or lobular carcinoma, and 80% of all breast cancers are ductal carcinomas, 90% of which are E-cad positive [3,4,5]. We employed α_5_β_1_ integrin-targeted nanoparticles and effectively delivered iMEK to breast cancer cells preferentially when compared to healthy breast cells. The MEK inhibitor was far more effective on E-cad-positive cells, and confirmation of E-cad expression as an indicator of successful therapeutic effect further demonstrated the potential clinical translation of our DDS. With regard to metastasis, a key cellular process that contributes to dissemination of tumor cells from the primary site is the epithelial-to-mesenchymal transition (EMT). Although it has been shown that EMT is associated with decreased E-cadherin expression [7,8], it must be noted that the likelihood of metastasis can also be reduced by eliminating hyper-proliferative cells that form the metastatic niche [7]. The MEK inhibitor and doxorubicin in our DDS are anti-proliferative agents that can contribute to the reduction in metastases by virtue of eliminating primary tumor cells at the local injection site.

The biodegradable, thermosensitive hydrogel utilized in this study has been proven to be safe for use and does not illicit a significant immune response on its own, as assessed by H&E assessment of foamy macrophages and neutrophils around the gel implant site (Figure 3). There was also no evidence of toxicity in RES organs, as determined by pathological assessment in either mouse study (biodegradation of empty hydrogel or implantation of empty hydrogel at the tumor site). 

To compare our DDS to other methods of delivery, we designed an orthotopic xenograft study of MDA-MB-231 E-cad+ TNBC in *NSG* mice. Compared to the controls (PBS and Gel), the Gel-NP(iMEK+DOX) system doubled both the lifetime and median survival time of mice (Figure 4c) and significantly minimized primary tumor growth (Figure 4e,f) and lung metastasis (Figure 4j). This observation contrasts with a pilot study where clinically relevant oral delivery of iMEK demonstrated significant toxicity—with two animals not surviving the entirety of the treatment plan—and only achieved marginal benefit in survival time that was not statistically significant compared to the DMSO control (Figure 4a). Importantly, oral iMEK delivery required a total dose of 300 mg/kg compared to a total dose of 4.5 mg/kg given for the nanoparticle system. In comparison to the other clinically relevant controls, I.V. nanoparticle injection with the Gel-NP(iMEK+DOX) system was able to extend median survival from 24 to 40 days when delivering the same amount of drug in three injections as opposed to five. Interestingly, all animals given the Gel-NP(iMEK+DOX) treatment had small tumors that did not require animals to be sacrificed, but they had skin ulcerations, which is one of the criteria for euthanasia. Further investigation is required to ascertain why these ulcers developed despite stabilization of tumor progression. Animals were observed scratching at the tumor site toward the end of the study, which could be a potential cause of the skin ulcerations. 

Our hydrogel-nanoparticle system was further evaluated in a *BALB/c* syngeneic mouse model of 4T1-luc IDC. The Gel-NP(iMEK+DOX) treatment increased median survival by a factor of approximately 2 (Figure 5b), similar to the *NSG* mouse model (Figure 4c), and resulted in long-term survival due to a halt in tumor progression for 4 out of 9 mice (Figure 5b,d,e). As in the case of the *NSG* mouse model, the animals did not succumb to the tumor but rather to skin ulcerations that could have been caused by animal scratching at the tumor site. Evaluation of the tumor microenvironment at the conclusion of the animal study revealed that the localized treatment of DOX and iMEK resulted in anticipated impacts on proliferation marker Ki67 and apoptotic marker cleaved caspase 3. We observed large areas of cleaved caspase 3-positive cells at the tumor edge, likely due to the peri-tumoral hydrogel injections, as this pattern of staining was absent in the two control groups (PBS and empty gel) (Figure 5j and Figure S5g). The increase in apoptotic cells at the tumor edge could explain the significant decrease in lung metastasis (Figure 5h), as fewer cells could successfully disseminate from the tumor, the initiation of the metastatic cascade. Thus, the tumor could not expand into neighboring tissues, reducing access to blood and lymphatic vessels. Analysis of the immune cell populations at the end of the study demonstrated that there were no statistically significant differences between the different conditions in the number of macrophages, neutrophils, dendritic cells, B cells (Figure S7), and the total T cell (Figure 5k) population. The decrease in the number of CD8+ cells (Figure 5m) at the end of the study could potentially be due to T cell exhaustion, as shown previously in 4T1 TNBC and GL261 glioblastoma mouse models [67,68]. Several groups have shown the role of increased CD4+ populations in long-term survivors in studies of 4T1 TNBC, B16F10 melanoma, and CT26 colon cancer mouse models, which is in agreement with our results (Figure 5l) [67,69,70]. It has also been shown that NK cells play an active role in extended survival in 4T1 TNBC, B16 melanoma, and CT26 colon cancer mouse models [71,72,73]. Therefore, the significant increase in CD4+ T cell (Figure 5l) and NK cell (Figure 5n) populations in our Gel-NP(iMEK+DOX) case could potentially explain the presence of long-term survivors. Future work should investigate the immune response further at earlier time points in the study, as well as probe the activation signatures of relevant immune cell populations. 

Clinical translation of this system also requires an assessment of how this hydrogel system can be incorporated into current standard-of-care mastectomy or lumpectomy surgery. IDC breast cancer treatment plans center around tumor excision, with neo-adjuvant therapies if possible [43]. Our dual-therapy hydrogel-nanoparticle system could be employed as a neo-adjuvant therapy to reduce breast tumors to a safe size prior to surgery for large lesions, or as an adjuvant therapy administered after surgery to ensure any cancer cells left behind are destroyed. Collectively, our work here demonstrates the therapeutic potential for local hydrogel administration to improve the therapeutic index of toxic drugs, reduce systemic toxicity, and reduce metastasis, all while improving survival outcomes. 

## Figures and Tables

**Table 1:** Primers used in the qPCR study.

Primer	Sequence
18s Human FWD	AGAAGTGACGCAGCCCTCTA
18s Human RVS	GAGGATGAGGTGGAACGTGT
18s Mouse FWD	CCGGCGACGACCCATTCGAAC
18s Mouse RVS	GAATCGAACCCTGATTCCCCGT
aTubulin Human FWD	AGGAGTCCAGATCGGCAATG
aTubulin Human RVS	GTCCCCACCACCAATGGTTT
aTubulin Mouse FWD	CACACAAGCTCACTCACCCT
aTubulin Mouse RVS	CTGTTATTAGGGATGTGACTCCA
GAPDH Human FWD	GGAGCGAGATCCCTCCAAAAT
GAPDH Human RVS	GGCTGTTGTCATACTTCTCATGGA
GAPDH Mouse FWD	TCACCACCATGGAGAAGGC
GAPDH Mouse RVS	GCTAAGCAGTTGGTGGTGCA
HK2 (Human) FWD	CCAGTTCATTCACATCATCAG
HK2 (Human) RVS	CTTACACGAGGTCACATAGC

**Table 2:** Antibodies used to stain different immune cells.

Antigen	Fluorophore	Biolegend Catalog Number
CD45	APC/Fire 750	157610
CD11b	FITC	101205
CD11c	PE/Cy7	117317
CD3	Alexa Fluor 700	100215
CD4	Spark UV™ 387	100491
CD8	BV510	100751
CD161	BV711	108745
F4/80	BV605	123133
CD19	BV785	115543
Ly6G	PE/Dazzle 594	127647
CD206	PE	141705
CD86	APC	105011
Live/Dead	Zombie Violet	423113

## Data Availability

All data needed to evaluate the conclusions are present in the paper and the Supplementary Materials.

## Supplementary Materials

The following supporting information can be downloaded at: https://www.mdpi.com/article/10.3390/pharmaceutics16080981/s1, Molecular dynamic simulations of BAB triblock copolymer self-assembly; **Figure S1**: (**a**) Survival plot based on Metabric data set [5]. Low E-cad expression is defined as 50 percentile or lower, while high E-cad expression is 50 percentile and above. N = 1902 patients. (**b**) Maximum-intensity projections of confocal microscopy images of live-dead signal in E-cad+ and E-cad− organoids, different z heights are represented in Figure 1. Scale bar = 200 μm, inset = 50 μm. (**c**) Cell proliferation of organoids assessed via PrestoBlue on days 1, 4, 7. Data are presented as mean ± SEM (*n* = 3 in quintuplicate). Statistical significance was determined using a two-sided unpaired *t* test. (**d**) α_5_β_1_ integrin expression on MDA-MB-231 organoids. Statistical significance was determined using a two-sided unpaired *t* test. (**e**) Cryo-TEM image of PR_b liposomes encapsulating iMEK+DOX. Scale bar = 200 nm. (**f**) MDA-MB-231 monolayer uptake experiment of PR_b functionalized liposomes and non-targeting liposomes. Statistical significance was determined using a two-sided unpaired *t* test. (**g**) 184B5 healthy mammary cell uptake of liposomes. **Figure S2:** (**a**) Cell viability of MDA-MB-231 E-cad+ and E-cad− organoids with PR_b functionalized liposomes and non-targeted liposomes loaded with various concentrations of iMEK and DOX. (**b**) E-cad+ organoids sustained release results for intermediate measurements (organoid 2 and 3) to complete data shown in Figure 2. (**c**) E-cad− organoid sustained release results for all organoid batches. **Figure S3:** Liver, spleen, and kidney H&E from various time points throughout the hydrogel biodegradation study, scale bar is 250 μm. **Figure S4**: (**a**) Mouse weights during the iMEK oral delivery study, demonstrating early toxicity effects. (**b**)Tumor weight of all groups after excision. Results are presented as mean ± SEM (*n* = 7–10) and statistical significance determined via one-way ANOVA with Tukey’s HSD post-hoc analysis; * *p* < 0.05, ** *p* < 0.01, *** *p* < 0.001. (**c**) H&E and IHC for pERK and Ki67 staining of primary tumors, scale bar is 3 mm. (**d**) RES organ assessment (spleen, liver, kidney) via H&E staining, scale bar is 200 μm. (**e**) H&E staining of lung sections from all groups. Scale bar is 3 mm and inset scale bar is 100 μm. **Figure S5**: (**a**) Tumor volume progression over time, measured with calipers in an orthotopic 4T1-luc tumor model of TNBC. Data are presented as mean ± SEM (*n* = 7–9) and statistical significance determined via one-way ANOVA with Tukey’s HSD post-hoc analysis; * *p* < 0.05, ** *p* < 0.01, *** *p* < 0.001. (**b**) Radiance images of tumors from Gel-NP (iMEK+DOX) group. (**c**) H&E staining of tumors from different treatment groups. Scale bar = 3 mm, inset = 100 μm. (**d**) RES organ assessment via H&E staining of kidney, liver, spleen, and heart for all groups, scale bar is 250 μm. (**e**) Weight of spleens from different treatment groups. Statistical significance determined via one-way ANOVA with Tukey’s HSD post-hoc analysis; * *p* < 0.05, ** *p* < 0.01, *** *p* < 0.001. (**f**) IHC staining of tumors for Ki67. Scale bar = 3 mm. (**g**) IHC staining of tumors for cleaved caspase 3. Scale bar = 3 mm. **Figure S6:** Gating strategy for quantifying immune cells in 4T1 tumors. **Figure S7**: Flow cytometric quantification of (**a**) macrophages, (**b**) CD206+ macrophages, (**c**) CD86+ macrophages, (**d**) dendritic cells, (**e**) neutrophils and (**f**) B cells. **Table S1:** Size and zeta potential of liposomes in HEPES buffer as determined by a Zetasizer. **Table S2:** Encapsulation efficiency (EE) of iMEK and DOX loaded in liposomes. References [5,31,74,75,76] are cited in the supplementary materials.
