# Supplementary material for: Local, Sustained, and Targeted Co-Delivery of MEK Inhibitor and Doxorubicin Inhibits Tumor Progression in E-Cadherin-Positive Breast Cancer
Source: Pharmaceutics. 2024 Jul 25;16(8):981. doi: 10.3390/pharmaceutics16080981 (PMC11357614; doi:10.3390/pharmaceutics16080981)
Supplement: Supplementary file 1 [file pharmaceutics-16-00981-s001.zip › pharmaceutics-3101645-supplementary.pdf]

pharmaceutics

Supplementary Materials

Local, Sustained, and Targeted Co-Delivery of MEK Inhibitor and Doxorubicin Inhibits Tumor Progression in E-Cadherin-Positive Breast Cancer

Paul M. Kuhn, Gabriella C. Russo, Ashleigh J. Crawford, Aditya Venkatraman, Nanlan Yang, Bartholomew A. Starich, Zachary Schneiderman, Pei-Hsun Wu, Thi Vo, Denis Wirtz and Efrosini Kokkoli

Molecular dynamic simulations of BAB triblock copolymer self-assembly

Simulation of the BAB triblock copolymer are performed using the HOOMD-Blue simulation engine [1]. We employ a coarse-grained mode of spherical beads of size σ connected via the standard FENE bond potential. To model the aggregation of the B-block into the micelle core, we assign an attractive Lennard-Jones (LJ) [2] interaction between all non-bonded beads associated with the B-block type within the system. All other non-bonded monomers interact via steric repulsion, modeled using the Weeks-Chandler-Andersen (WCA) potential [3]. To model PEG-swelling/shrinkage due to dehydration (A-block) with rising temperatures, WCA repulsions between A-block type particles are defined to decrease with increasing system temperature. Similarly, decreased hydration results in stronger hydrophobic interactions between A and B particles, thus AB WCA repulsions also increase with increasing system temperature. We vary both the simulation temperature (kT) and chain length ratio N_B/N_{BA}. Temperatures are sampled for $kT \in [0.25, 10]$ and chain length ratio in terms of repeat units for $N_B/N_A \in [0.125, 2.0]$, totaling to approximately 85 state points across the sample kT and N_B/N_A phase space. All simulations consist of 625 BAB triblock polymer chains, resulting in system sizes ranging from 15,625 to 62,500 particles, depending on the N_B/N_A ratio. All simulations are performed in the NVT ensemble with an integration step size $dt = 0.001$.

Each simulation starts with chains initialized in a 25x25 grid (along the X-by-Y direction), with initial velocities sampled from a distribution defined by the system temperature kT. The system is equilibrated over 10^6 timesteps, where all non-bonded interactions are initially purely repulsive. After equilibration, the system is slowly compressed to a target volume fraction of ~15% over 10^6 timesteps, followed by an additional 10^6 timesteps to equilibrate the system at the target density. B-B interactions are then switched to LJ (attractive) and the simulation is then ran for a production run of 10^7 timesteps.

Characterization of the micelle structures are performed by first clustering particles into their respective micelles. Note that, for cases where all particles are connected into a network worm-like micelle, the number of clusters is one. For each cluster, we then compute the diagonalized gyration tensor for the core monomers (B-block). Micelles with symmetric tensors are defined as spherical. Micelles with asymmetric tensors, where one dimension spans longer than half the box length are defined as worm-like. For rapid clustering and computation of the gyration tensors, we employ the *freud* analysis package [4]. All quantities are averaged over the second half of the production run (timesteps from $5x10^6$ to 10^7) and averaged over three independent runs.

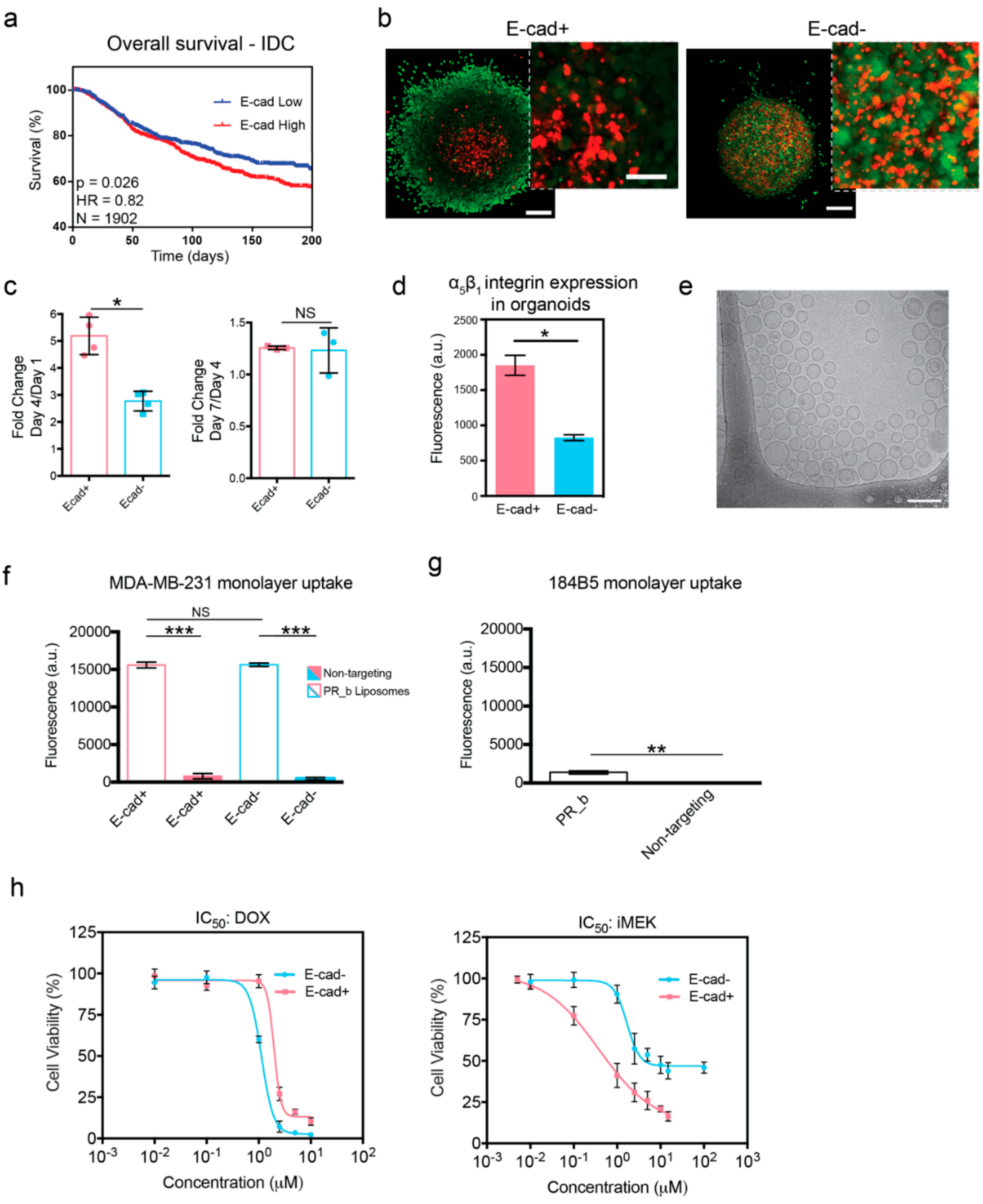

Figure S1. (**a**) Survival plot based on Metabric data set [5]. Low E-cad expression is defined as 50 percentile or lower, while high E-cad expression is 50 percentile and above. N = 1902 patients. (**b**) Maximum-intensity projections of confocal microscopy images of live-dead signal in E-cad+ and E-cad- organoids, different z heights are represented in Figure 1. Scale bar = 200 μm, inset = 50 μm. (**c**) Cell proliferation of organoids assessed via PrestoBlue on days 1, 4, 7. Data are presented as mean ± SEM (n = 3 in quintuplicate). Statistical significance was determined using a two-sided unpaired t test. (**d**) $\alpha_5\beta_1$ integrin expression on MDA-MB-231 organoids. Statistical significance was determined using a two-sided unpaired t test. (**e**) Cryo-TEM image of PR_b liposomes encapsulating iMEK+DOX. Scale bar = 200 nm. (**f**) MDA-MB-231 monolayer uptake experiment of PR_b functionalized liposomes and non-targeting liposomes. Statistical significance was determined using a two-sided unpaired t test. (**g**) 184B5 healthy mammary cell uptake of liposomes. Statistical significance was determined using a two-sided unpaired t test. (**h**) IC_{50} of DOX and iMEK in both E-cad+ and E-cad-organoids. Data are presented as mean ± SEM (n = 3). For all plots in this figure; * $p < 0.05$, ** $p < 0.01$, *** $p < 0.001$.

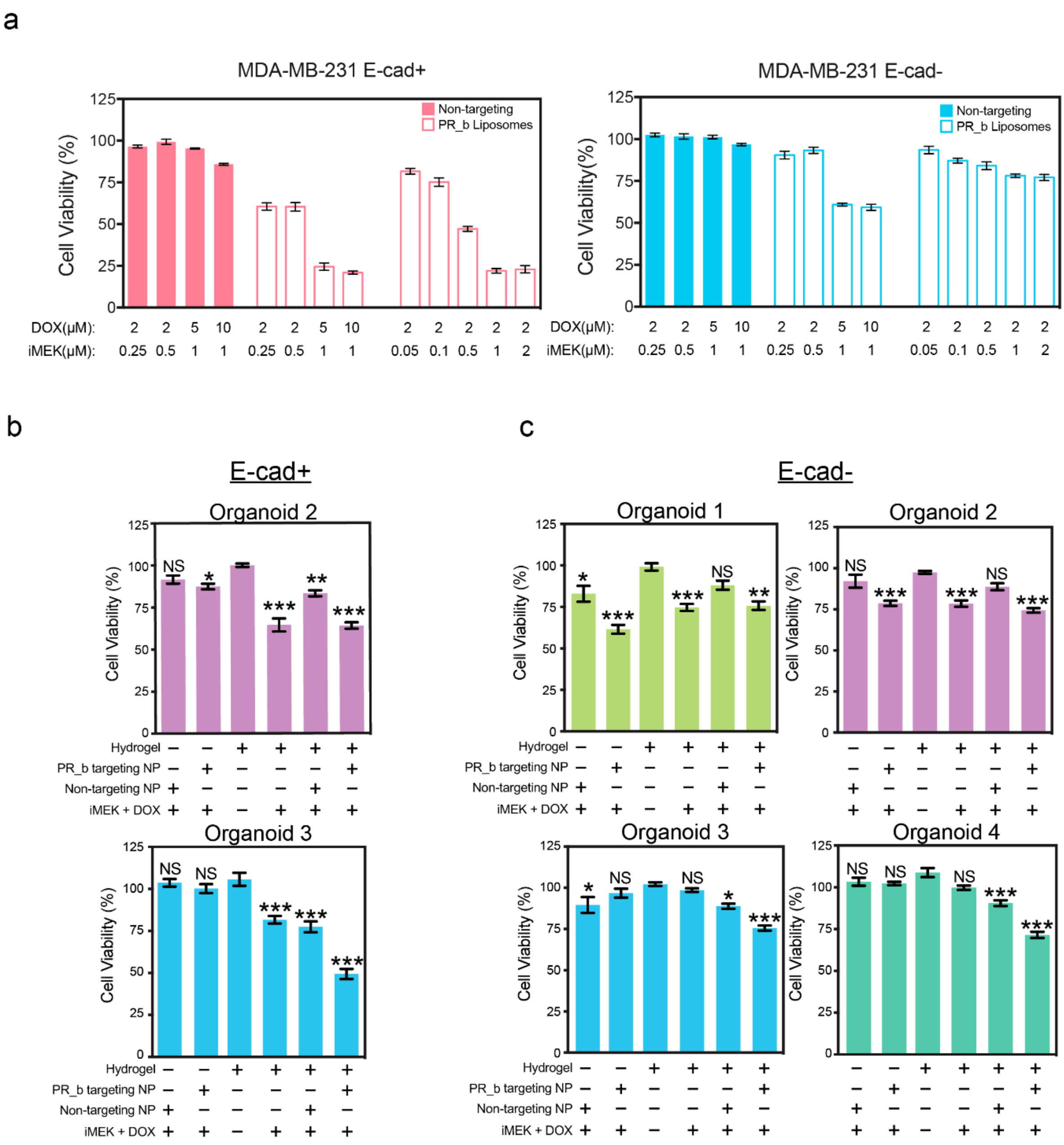

Figure S2. (**a**) Cell viability of MDA-MB-231 E-cad+ and E-cad- organoids with PR_b functionalized liposomes and non-targeted liposomes loaded with various concentrations of iMEK and DOX. (**b**) E-cad+ organoids sustained release results for intermediate measurements (organoid 2 and 3) to complete data shown in Figure 2. (**c**) E-cad- organoid sustained release results for all organoid batches. For all plots in this figure data are presented as mean ± SEM (n = 3). Statistical significance determined via one-way ANOVA with Tukey's HSD post-hoc analysis; * $p < 0.05$, ** $p < 0.01$, *** $p < 0.001$.

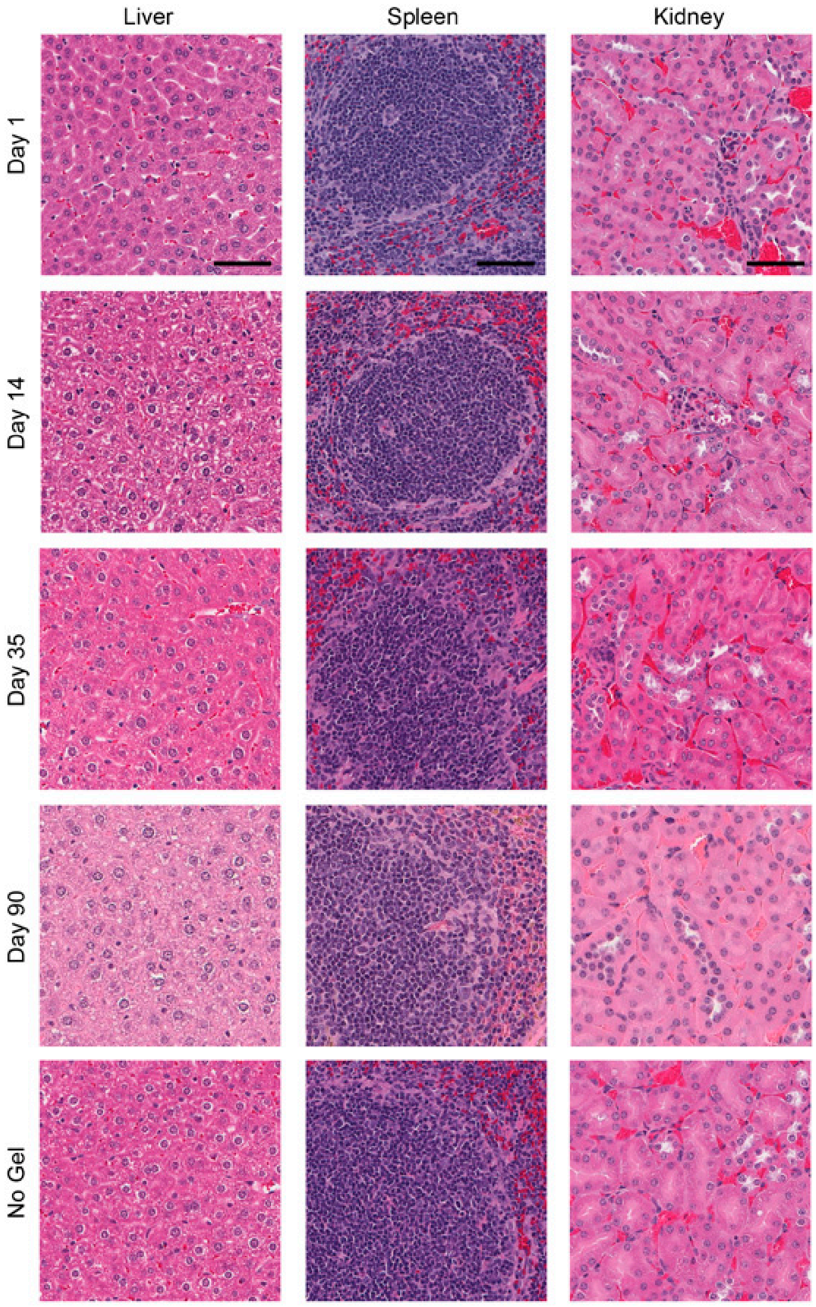

Figure S3. Liver, spleen, and kidney H&E from various time points throughout the hydrogel biodegradation study, scale bar is 250 µm.

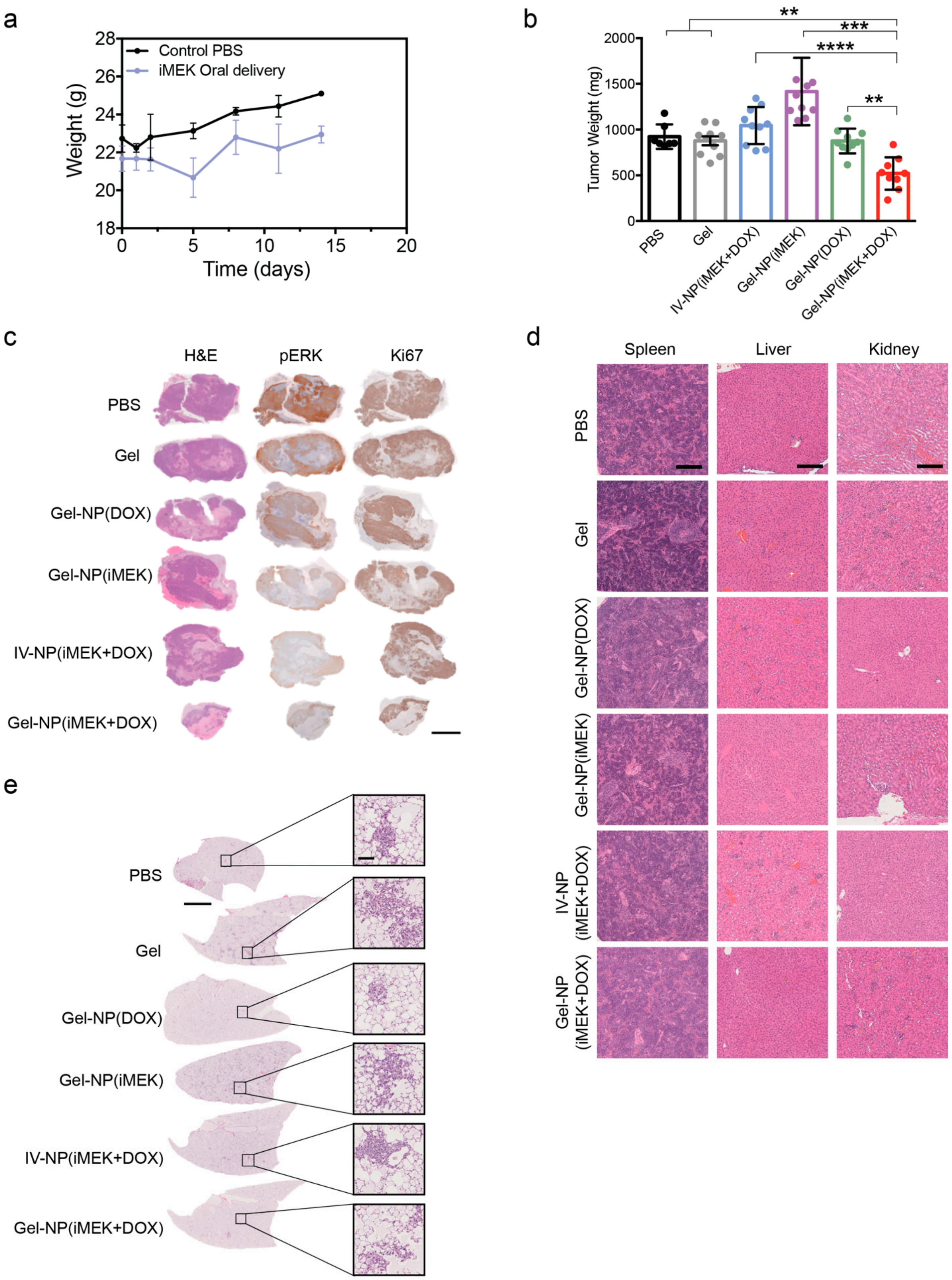

Figure S4. (**a**) Mouse weights during the iMEK oral delivery study, demonstrating early toxicity effects. (**b**)Tumor weight of all groups after excision. Results are presented as mean ± SEM (n = 7–10) and statistical significance determined via one-way ANOVA with Tukey's HSD post-hoc analysis; * $p < 0.05$, ** $p < 0.01$, *** $p < 0.001$. (**c**) H&E and IHC for pERK and Ki67 staining of primary tumors, scale bar is 3 mm. (**d**) RES organ assessment (spleen, liver, kidney) via H&E staining, scale bar is 200 µm. (**e**) H&E staining of lung sections from all groups. Scale bar is 3 mm and inset scale bar is 100 µm. All tissues were harvested upon animal termination criteria.

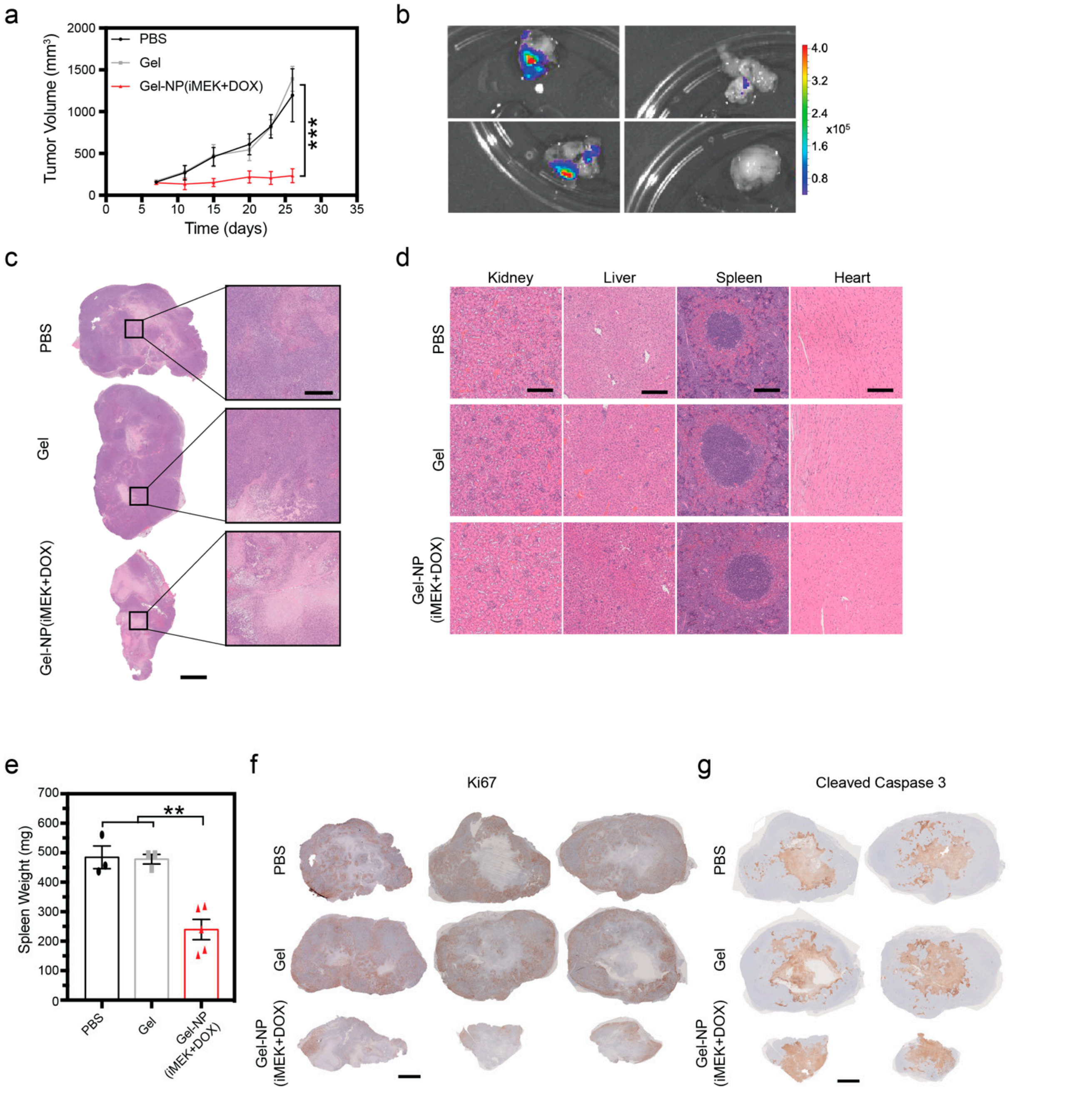

Figure S5. (**a**) Tumor volume progression over time, measured with calipers in an orthotopic 4T1-luc tumor model of TNBC. Data are presented as mean ± SEM (n = 7–9) and statistical significance determined via one-way ANOVA with Tukey's HSD post-hoc analysis; * $p < 0.05$, ** $p < 0.01$, *** $p < 0.001$. (**b**) Radiance images of tumors from Gel-NP(iMEK+DOX) group. (**c**) H&E staining of tumors from different treatment groups. Scale bar = 3 mm, inset = 100 µm. (**d**) RES organ assessment via H&E staining of kidney, liver, spleen, and heart for all groups, scale bar is 250 µm. (**e**) Weight of spleens from different treatment groups. Statistical significance determined via one-way ANOVA with Tukey's HSD post-hoc analysis; * $p < 0.05$, ** $p < 0.01$, *** $p < 0.001$. (**f**) IHC staining of tumors for Ki67. Scale bar = 3 mm. (**g**) IHC staining of tumors for cleaved caspase 3. Scale bar = 3 mm. All tissues were harvested upon animal termination criteria or end of the study.

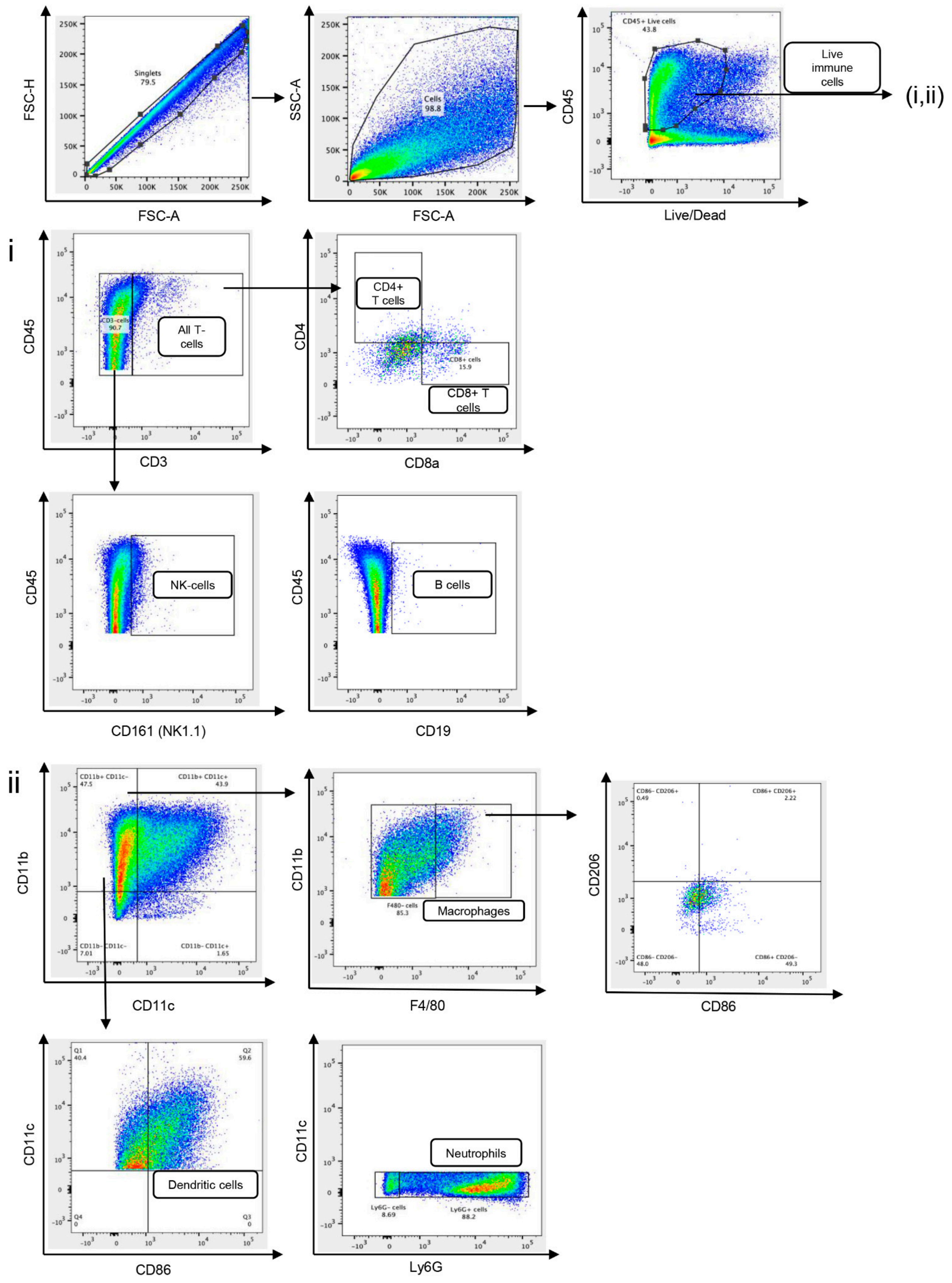

Figure S6. Gating strategy for quantifying immune cells in 4T1 tumors.

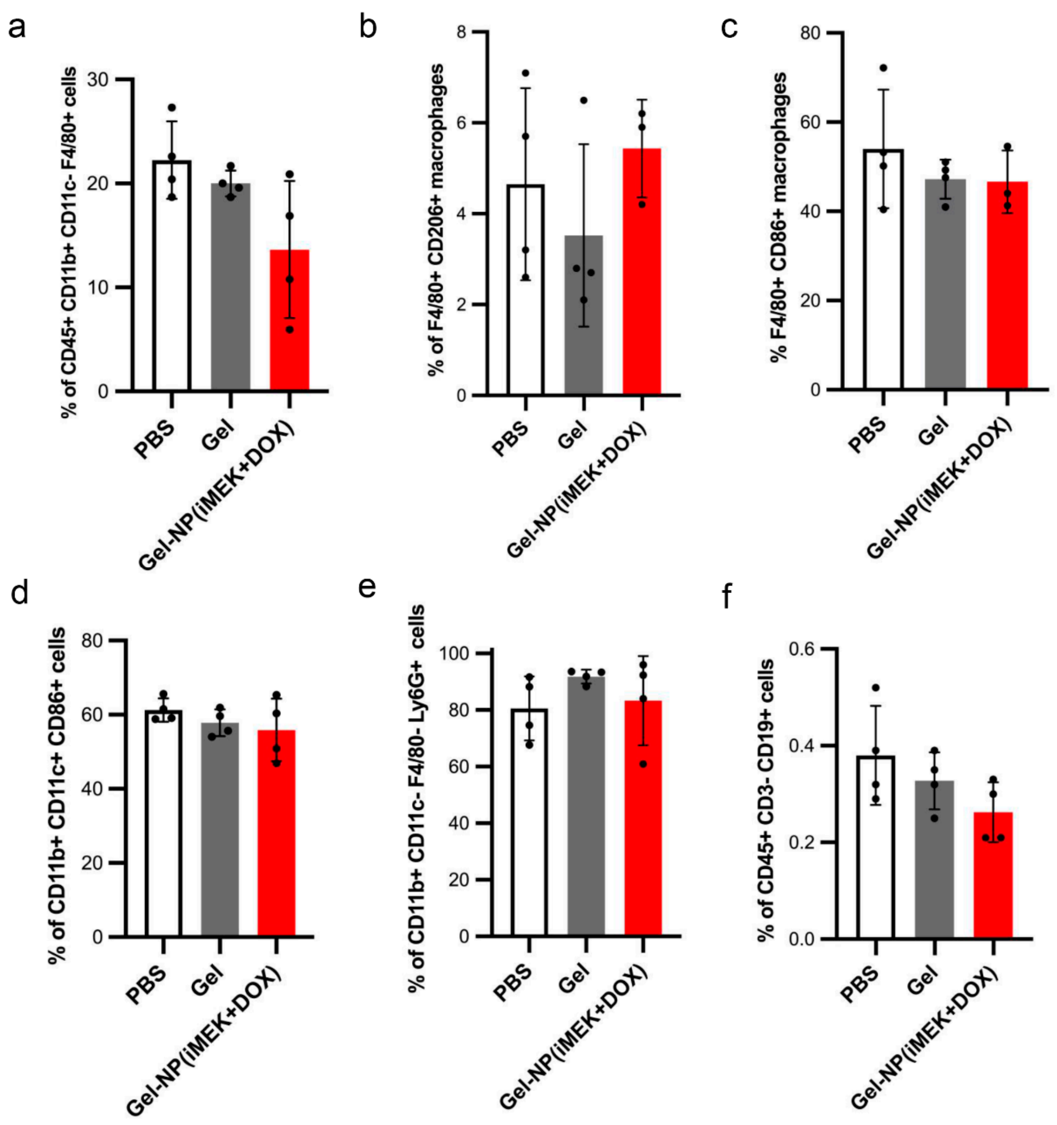

Figure S7. Flow cytometric quantification of (**a**) macrophages, (**b**) CD206+ macrophages, (**c**) CD86+ macrophages, (**d**) dendritic cells, (**e**) neutrophils and (**f**) B cells. Statistical significance was determined via one-way ANOVA with Tukey's HSD post-hoc analysis. For all pairs in this figure, $p > 0.05$.

Table S1. Size and zeta potential of liposomes in HEPES buffer as determined by a Zetasizer.

Formulation	Size (nm)	Zeta Potential (mV)
Empty Liposomes	118 ± 1.8	-14.1 ± 1.1
DOX Liposomes	123 ± 2.3	-13.4 ± 1.3
iMEK Liposomes	117 ± 2.1	-12.4 ± 0.3
iMEK+DOX Liposomes	123 ± 1.3	-12.6 ± 1.7
iMEK PR_b Liposomes	125 ± 2.6	11.5 ± 1.5
DOX PR_b Liposomes	124 ± 1.6	12.2 ± 1.0
iMEK+DOX PR_b Liposomes	126 ± 2.3	12.2 ± 0.8

Table S2. Encapsulation efficiency (EE) of iMEK and DOX loaded in liposomes.

Formulation	%EE of iMEK	%EE of DOX
DOX Liposomes		96.7 ± 1.1
iMEK Liposomes	76.1 ± 3.1	
iMEK+DOX Liposomes	78.3 ± 1.1	95.4 ± 2.6
iMEK PR_b Liposomes	76.4 ± 2.2	
DOX PR_b Liposomes		95.3 ± 2.2
iMEK+DOX PR_b Liposomes	74.4 ± 2.8	97.1 ± 3.2

References

1. Anderson, J.A.; Glaser, J.; Glotzer, S.C. HOOMD-blue: A Python package for high-performance molecular dynamics and hard particle Monte Carlo simulations. *Computational Materials Science* **2020**, *173*, 109363. [https://10.1016/j.commatsci.2019.109363]
2. Wood, W.W.; Parker, F.R. Monte Carlo Equation of State of Molecules Interacting with the Lennard-Jones Potential. I. A Supercritical Isotherm at about Twice the Critical Temperature. *The Journal of Chemical Physics* **2004**, *27*, 720-733. [https://10.1063/1.1743822]
3. Weeks, J.D.; Chandler, D.; Andersen, H.C. Role of Repulsive Forces in Determining the Equilibrium Structure of Simple Liquids. *The Journal of Chemical Physics* **1971**, *54*, 5237-5247. [https://10.1063/1.1674820]
4. Ramasubramani, V.; Dice, B.D.; Harper, E.S.; Spellings, M.P.; Anderson, J.A.; Glotzer, S.C. freud: A software suite for high throughput analysis of particle simulation data. *Computer Physics Communications* **2020**, *254*, 107275. [https://10.1016/j.cpc.2020.107275]
5. Curtis, C.; Shah, S.P.; Chin, S.F.; Turashvili, G.; Rueda, O.M.; Dunning, M.J.; Speed, D.; Lynch, A.G.; Samarajiwa, S.; Yuan, Y.; Gräf, S.; Ha, G.; Haffari, G.; Bashashati, A.; Russell, R.; Mckinney, S.; Langerød, A.; Green, A.; Provenzano, E.; Wishart, G.; Pinder, S.; Watson, P.; Markowetz, F.; Murphy, L.; Ellis, I.; Purushotham, A.; Børresen-Dale, A.L.; Brenton, J.D.; Tavaré, S.; Caldas, C.; Aparicio, S. The genomic and transcriptomic architecture of 2,000 breast tumours reveals novel subgroups. *Nature* **2012**, *486*, 346-352. [https://10.1038/nature10983]
